# Enhanced skeletal muscle insulin sensitivity after acute resistance-type exercise is upregulated by rapamycin-sensitive mTOR complex 1 inhibition

**DOI:** 10.1038/s41598-020-65397-z

**Published:** 2020-05-22

**Authors:** Kohei Kido, Kohei Sase, Takumi Yokokawa, Satoshi Fujita

**Affiliations:** 1 Faculty of Sport and Health Science, Ritsumeikan University, Kusatsu, Shiga Japan; 2 Laboratory of Sports and Exercise Medicine, Graduate School of Human and Environmental Studies, Kyoto University, Kyoto, Japan

**Keywords:** Metabolism, Intracellular signalling peptides and proteins, Calcium and phosphate metabolic disorders

## Abstract

Acute aerobic exercise (AE) increases skeletal muscle insulin sensitivity for several hours, caused by acute activation of AMP-activated protein kinase (AMPK). Acute resistance exercise (RE) also activates AMPK, possibly improving insulin-stimulated glucose uptake. However, RE-induced rapamycin-sensitive mechanistic target of rapamycin complex 1 (mTORC1) activation is higher and has a longer duration than after AE. In molecular studies, mTORC1 was shown to be upstream of insulin receptor substrate 1 (IRS-1) Ser phosphorylation residue, inducing insulin resistance. Therefore, we hypothesised that although RE increases insulin sensitivity through AMPK activation, prolonged mTORC1 activation after RE reduces RE-induced insulin sensitising effect. In this study, we used an electrical stimulation–induced RE model in rats, with rapamycin as an inhibitor of mTORC1 activation. Our results showed that RE increased insulin-stimulated glucose uptake following AMPK signal activation. However, mTORC1 activation and IRS-1 Ser632/635 and Ser612 phosphorylation were elevated 6 h after RE, with concomitant impairment of insulin-stimulated Akt signal activation. By contrast, rapamycin inhibited these prior exercise responses. Furthermore, increases in insulin-stimulated skeletal muscle glucose uptake 6 h after RE were higher in rats with rapamycin treatment than with placebo treatment. Our data suggest that mTORC1/IRS-1 signaling inhibition enhances skeletal muscle insulin-sensitising effect of RE.

## Introduction

A single bout of exercise, especially aerobic exercise (AE), increases the effect of insulin on skeletal muscle glucose uptake^1–9^. This phenomenon is observed in both human and rodent skeletal muscle and may be sustained for up to 48 h after exercise^10–12^. Because skeletal muscle accounts for up to 85% of insulin-stimulated whole-body glucose uptake^13,14^, this observation is important for blood glucose control. Previous studies, using skeletal muscle-specific AMP-activated protein kinase (AMPK) α1 and α2 double-knockout mice, indicated that the insulin-sensitising effect of prior AE was due to energy stress-sensitive AMPK activation^15,16^. The previous studies^15–17^ and our study^18^ showed that a downstream target of both AMPK and insulin pathways, TBC1 domain family member 4 (TBC1D4) phosphorylation at Thr649 and Ser711, facilitated the effect of acute AMPK activation to enhance glucose uptake in response to insulin. These studies suggested that the magnitude of AMPK pathway activation in response to prior exercise is an important factor for increasing skeletal muscle insulin sensitivity.

Resistance exercise (RE) is another exercise method, which causes skeletal muscle hypertrophy in both humans and rodents. Although previous studies showed the effect of a single bout of RE on whole-body insulin sensitivity or glucose tolerance^19–28^, quantitative assessment of skeletal muscle glucose uptake has not yet been performed. Nevertheless, previous human studies suggested that acute RE increased AMPK activation and TBC1D4 Ser/Thr phosphorylation in skeletal muscle^29,30^. Moreover, we previously showed in a rodent RE model that there was increased AMPKα Thr172 phosphorylation and TBC1D4 Thr649 phosphorylation, both of which were prolonged more than that after AE^31,32^. Accordingly, acute RE might cause a further increase in insulin-stimulated skeletal muscle glucose uptake.

Mechanistic target of rapamycin complex 1 (mTORC1) activation is caused by acute RE^33^, which is higher than the effect seen after acute AE^34^, and that persists for up to 24 h in humans^35^. This activation promotes protein translation^33,36^, resulting in increased muscle protein synthesis and subsequent muscle hypertrophy^37^. Therefore, mTORC1 activation in response to RE is important for skeletal muscle hypertrophy. However, mTORC1 activation decreases insulin-stimulated glucose uptake in various insulin-sensitive tissues, respectively^38–41^; insulin resistance is explained by a negative feedback loop from mTORC1 activation to insulin receptor substrate 1 (IRS-1) Ser phosphorylation residues (e.g., Ser616, Ser636/639, and Ser1101 in human) that counteract with insulin-stimulated IRS-1 Try phosphorylation and following downstream signal activation (e.g., PI3K, Akt, and GLUT4 trafficking)^38–44^. According to these findings, prolonged mTORC1 pathway activation after acute RE may inhibit or disrupt increases in skeletal muscle insulin sensitivity, competing with AMPK pathway activation.

mTORC1 is one of the complexes of mTOR, and a subset of mTORC1-dependent, but not mTORC2-dependent, pathway activation is highly selective to inhibition by rapamycin^45^. Thus, it has been widely known that mTORC1 is responsible for the rapamycin-sensitive signaling events. For this reason, we used rapamycin to identify the effect of rapamycin-sensitive mTORC1 pathway activation on the insulin-sensitising effect of an acute bout of RE in rat skeletal muscle.

## Results

### AMPK pathway activation after acute RE

**Figure 1:**
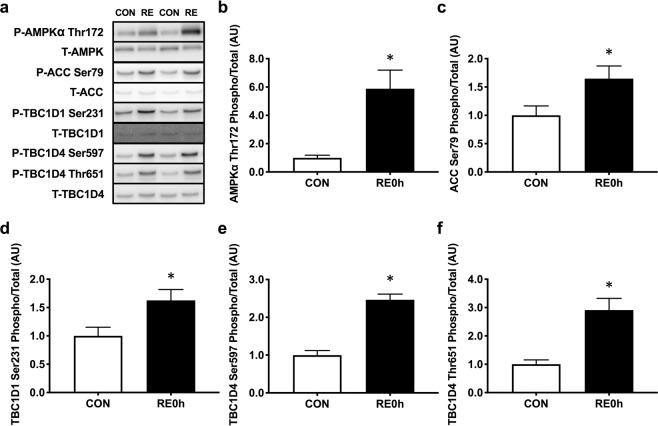
AMPK pathway activation immediately after RE. (**a**) Representative Western blot images. The grouping of blots cropped from different parts of the same gel, or from different gels, fields, or exposures were divided by black lines. (**b**) AMPKα Thr172, (**c**) ACC Ser79, (**d**) TBC1D1 Ser231, (**e)** TBC1D4 Ser597 and (**f**) TBC1D4 Thr651 phosphorylation in response to a single bout of RE. *n* = 5. Values are means ± standard error. *P < 0.05. RE, resistance exercise; CON, unstimulated control.

To confirm positive signal activation for insulin sensitivity, we measured AMPK pathway activation immediately after RE (Fig. 1a–f). We found that acute RE significantly increased phosphorylation of AMPKα Thr172 (Fig. 1b) and the downstream targets acetyl-CoA carboxylase (ACC) Ser79 (Fig. 1c), TBC1 domain family member 1 (TBC1D1) Ser231 (Fig. 1d), TBC1D4 Ser597 (Fig. 1e) and TBC1D4 Thr651 (Fig. 1f). Collectively, these results implicate that acute RE possibly improves insulin sensitivity because of AMPK pathway activation.

### mTORC1 activation and IRS-1 Ser phosphorylation by acute RE

**Figure 2:**
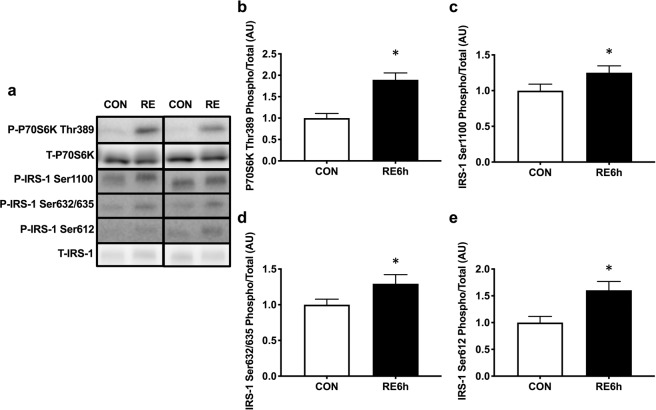
mTORC1-IRS-1 pathway activation 6 h after RE. (**a**) Representative Western blot images. The grouping of blots cropped from different parts of the same gel, or from different gels, fields, or exposures were divided by black lines. (**b)** P70S6K Thr389, (**c**) IRS-1 Ser1100, (**d**) IRS-1 Ser632/635 and (**e**) IRS-1 Ser612 phosphorylation in response to a single bout of RE. *n* = 12. Values are means ± standard error. *P < 0.05. RE, resistance exercise.

A marker of mTORC1 activation, ribosomal protein S6 kinase (P70S6K) phosphorylation^46^, remained elevated 6 h after RE (Fig. 2a,b). IRS-1 Ser1100 (Fig. 2a,c), Ser632/635 (Fig. 2a,d), and Ser612 (Fig. 2a,e) were also increased by RE.

### Effect of a prior bout of RE on insulin sensitivity

**Figure 3:**
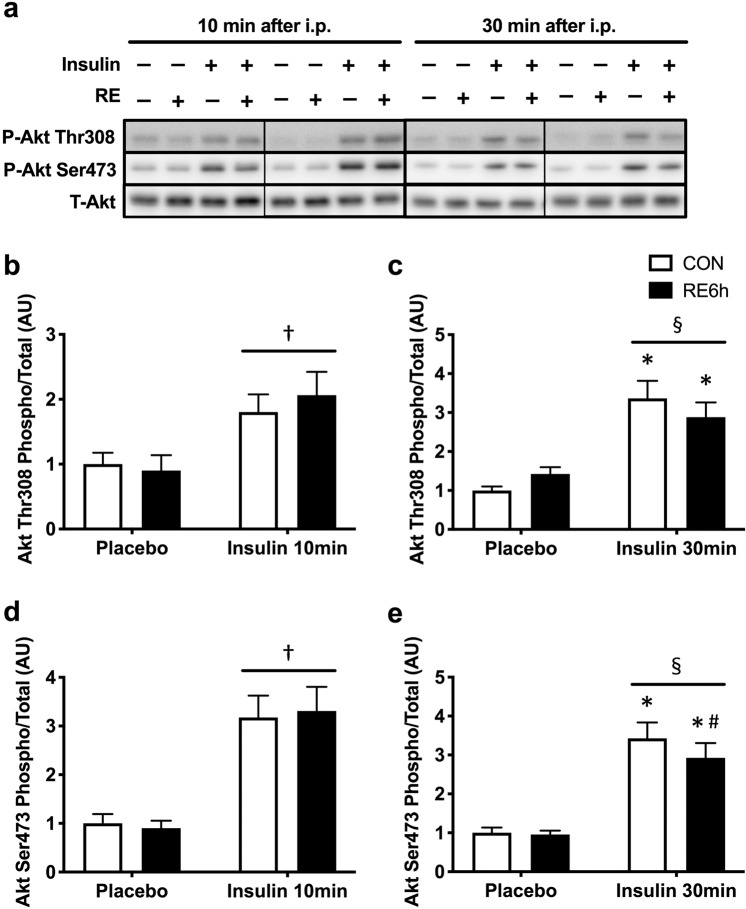
Akt phosphorylation in response to insulin 6 h after RE. (**a**) Representative Western blot images. The grouping of blots cropped from different parts of the same gel, or from different gels, fields, or exposures were divided by black lines. (**b**) Akt Thr308 and (**d**) Akt Ser473 phosphorylation 10 min after insulin injection. **(c**) Akt Thr308 and (**e**) Akt Ser473 phosphorylation 30 min after insulin injection. *n* = 6 in each group. Values are means ± standard error. *P < 0.05 versus placebo injection within CON or RE legs, ^♯^P < 0.05 versus CON leg for each group, ^†^P < 0.05 main effect of insulin, ^§^P < 0.05 versus response to insulin (interaction of insulin × RE). RE, resistance exercise; CON, unstimulated control.

**Figure 4:**
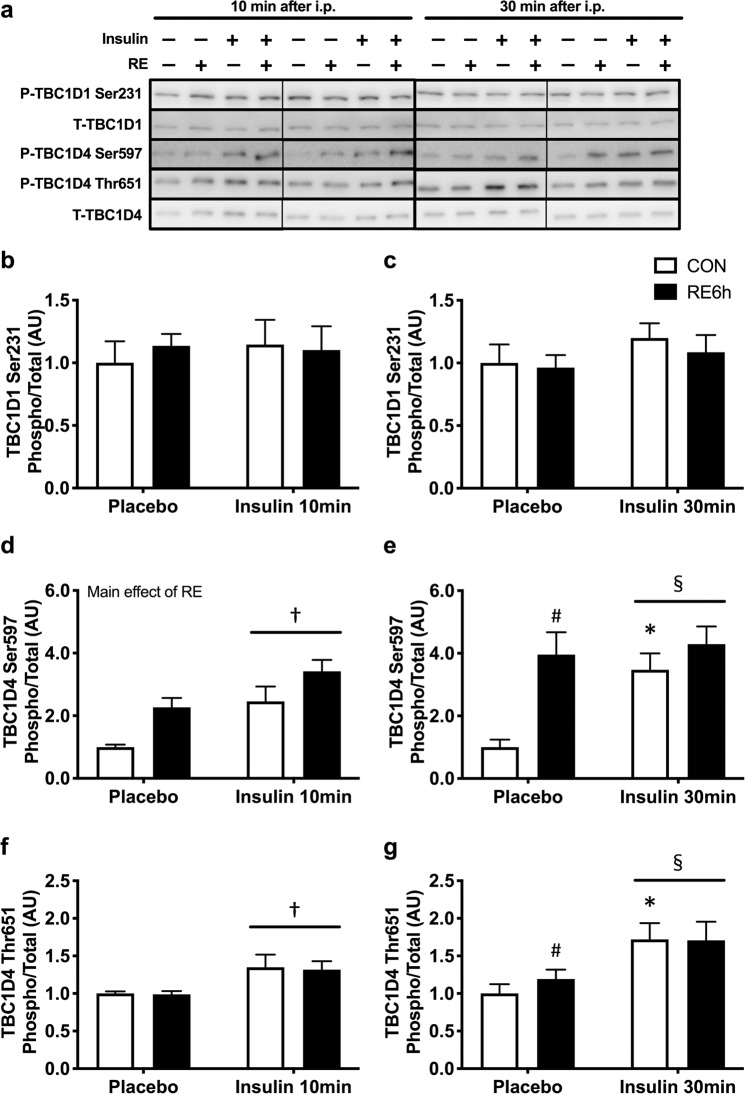
TBC1D1/TBC1D4 phosphorylation in response to insulin 6 h after RE. (**a**) Representative Western blot images. The grouping of blots cropped from different parts of the same gel, or from different gels, fields, or exposures were divided by black lines. (**b**) TBC1D1 Ser231, (**d**) TBC1D4 Ser597 and (**f)** TBC1D4 Thr651 phosphorylation 10 min after insulin injection. (**c**) TBC1D1 Ser231, (**e)** TBC1D4 Ser597 and (**g)** TBC1D4 Thr651 phosphorylation 30 min after insulin injection. *n* = 6 in each group. Values are means ± standard error. *P < 0.05 versus placebo injection within CON or RE legs, ^♯^P < 0.05 versus CON leg for each group, ^†^P < 0.05 main effect of insulin, ^§^P < 0.05 versus response to insulin (interaction of insulin × RE). RE, resistance exercise; CON, unstimulated control.

**Figure 5:**
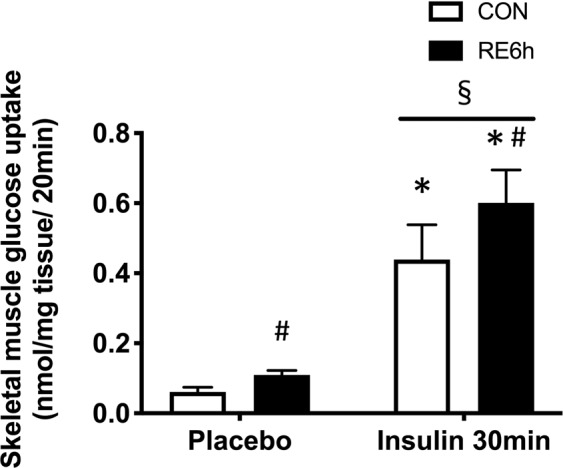
Skeletal muscle glucose uptake in response to insulin 6 h after RE. Skeletal muscle 2-deoxy-d-glucose uptake 30 min after insulin injection. *n* = 6–7 in each group. Values are means ± standard error. *P < 0.05 versus placebo injection within CON or RE legs, ^♯^P < 0.05 versus CON leg for each group, ^§^P < 0.05 versus response to insulin (interaction of insulin × RE). RE, resistance exercise; CON, unstimulated control.

Insulin stimulation for both 10 and 30 min significantly increased phosphorylation of Akt Thr308 (Fig. 3a–c), Akt Ser473 (Fig. 3a,d,e), TBC1D4 Ser597 (Fig. 4a,d,e) and TBC1D4 Thr651 (Fig. 4a,f,g), but not TBC1D1 Ser231 (Fig. 4a–c). Despite the absence of significant interaction between RE and 10-min insulin stimulation (Figs. 3b,d and 4d,f), prior RE significantly impaired 30-min insulin-stimulated Akt Ser473 (Fig. 3e), TBC1D4 Ser597 (Fig. 4e) and TBC1D4 Thr651 phosphorylation (Fig. 4g). Moreover, 30-min insulin-stimulated Akt Thr308 in the exercised leg showed an insignificant but similar trend as that of Akt Ser473 (p = 0.1) (Fig. 3c). These results suggest that prior RE causes skeletal muscle insulin resistance. By contrast, 30-min insulin stimulation significantly increased skeletal muscle glucose uptake, with further elevation in the exercised muscle (Fig. 5).

### Inhibition of rapamycin-sensitive mTORC1 pathway activation

**Figure 6:**
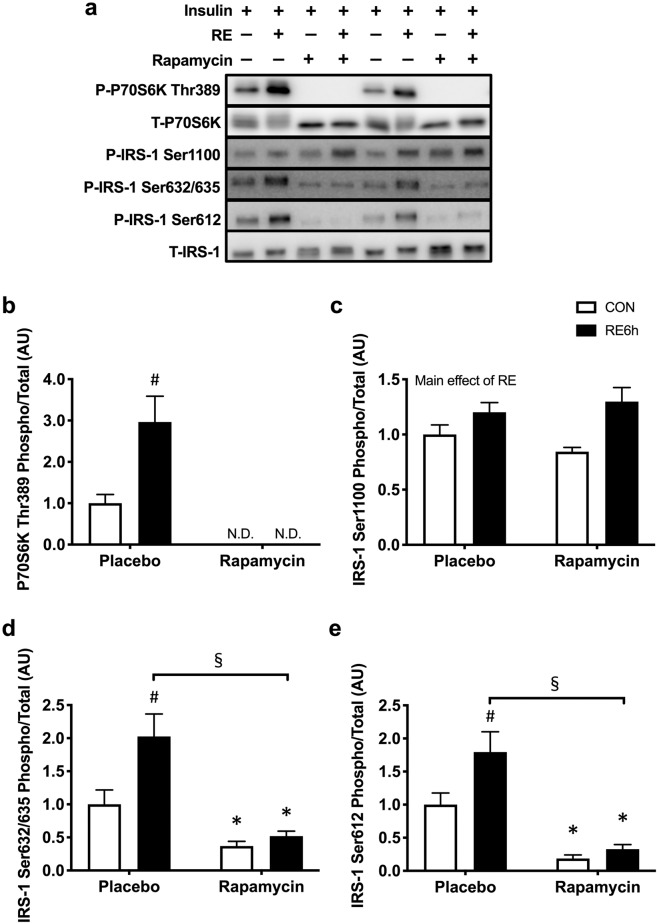
Effect of rapamycin on mTORC1/IRS-1 pathway activation by RE. (**a**) Representative Western blot images. The grouping of blots cropped from different parts of the same gel, or from different gels, fields, or exposures were divided by black lines. (**b**) P70S6K Thr389, (**c**) IRS-1 Ser1100, (**d**) IRS-1 Ser632/635 and (**e)** IRS-1 Ser612 phosphorylation in response to single bout of RE under treatment of either placebo or rapamycin. *n* = 4 in each group. Values are means ± standard error. *P < 0.05 versus placebo injection within CON or RE legs, ^♯^P < 0.05 versus CON leg for each group, ^§^P < 0.05 versus response to RE (interaction of RE × rapamycin). RE, resistance exercise; CON, unstimulated control.

Rapamycin completely inhibited P70S6K phosphorylation in both basal and exercised states (Fig. 6a,b). Moreover, phosphorylation of IRS-1 at Ser632/635 and Ser612 were lowered by rapamycin in the basal state, and the exercise effects on these phosphorylations were diminished (Fig. 6a,d,e). However, IRS-1 Ser1100 phosphorylation levels were not different between the placebo and rapamycin groups (Fig. 6a,c).

### Interaction between mTORC1/IRS-1 Ser pathway activation and insulin sensitivity after acute RE

**Figure 7:**
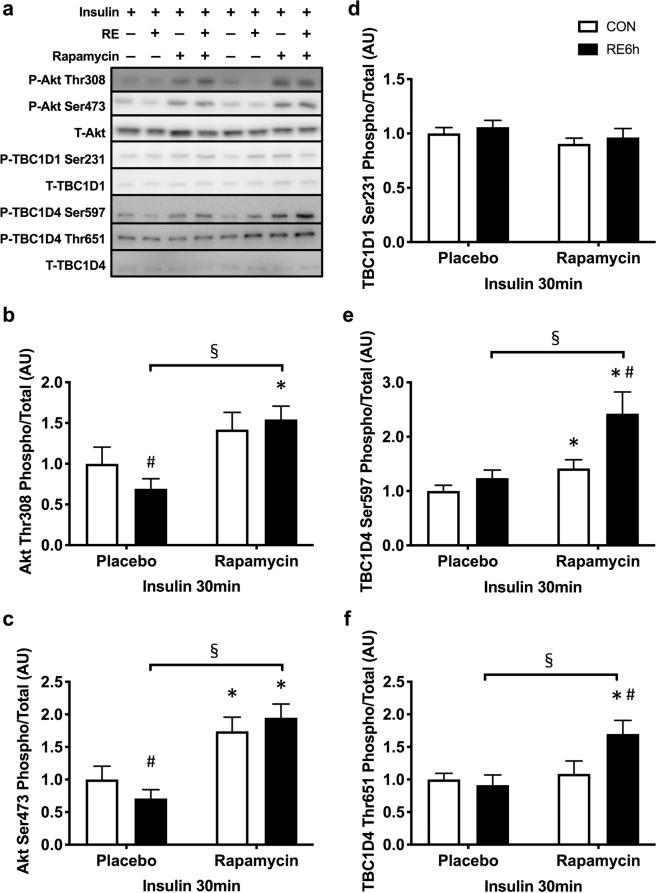
Rapamycin inhibits decease in insulin-stimulated Akt pathway activation in exercised leg. (**a**) Representative Western blot images. The grouping of blots cropped from different parts of the same gel, or from different gels, fields, or exposures were divided by black lines. (**b**) Akt Th308, (**c**) Akt Ser473, (**d**) TBC1D1 Ser231, (**e**) TBC1D4 Set597 and (**f**) TBC1D4 Thr651 phosphorylation in response to insulin in CON or RE leg under treatment of either placebo or rapamycin. *n* = 6–8 in each group. Values are means ± standard error. *P < 0.05 versus placebo injection within CON or RE legs, ^♯^P < 0.05 versus CON leg for each group, ^§^P < 0.05 versus response to RE (interaction of RE × rapamycin). RE, resistance exercise; CON, unstimulated control.

**Figure 8:**
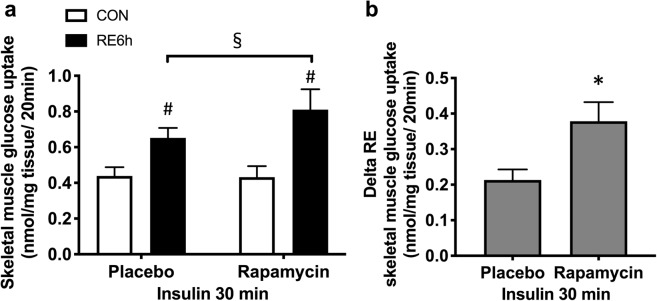
Rapamycin improves increase in skeletal muscle glucose uptake by RE. (**a**) Insulin-stimulated skeletal muscle 2-deoxy-d-glucose uptake in response to insulin in CON or RE leg under treatment of either placebo or rapamycin. (**b**) Delta increase in insulin-stimulated skeletal muscle 2-deoxy-d-glucose uptake by prior single bout of RE. *n* = 5–6 in each group. Values are means ± standard error. *P < 0.05 versus placebo injection, ^♯^P < 0.05 versus CON leg for each group, ^§^P < 0.05 versus response to RE (interaction of RE × rapamycin). RE, resistance exercise; CON, unstimulated control.

Insulin-stimulated TBC1D1 Ser231 phosphorylation was not affected by either prior RE or rapamycin (Fig. 7a,d). In the placebo group, insulin-stimulated Akt Thr308 and Ser473 phosphorylations were lowered by prior RE, and the impairment was totally reversed by rapamycin (Fig. 7a–c). Following these upstream responses, although phosphorylation of TBC1D4 Ser597 and Thr651 in response to insulin was not different between control and exercised legs in the placebo group, these phosphorylations were significantly higher in the exercised leg in the rapamycin group (Fig. 7a,e,f). In Fig. 4e,g, we showed an elevation of *p*-TBC1D4 Ser597 and Thr651 by RE without insulin. This may be the reason why insulin-stimulated *p*-TBC1D4 Ser597 and Thr651 levels were higher in the exercised leg in rapamycin-treated rats. Furthermore, the increase in skeletal muscle glucose uptake by exercise under insulin stimulating conditions was significantly improved by rapamycin (Fig. 8a,b). These results suggest that prior RE-induced mTORC1 activation and subsequent IRS-1 Ser phosphorylation lowered the improvement of insulin sensitivity.

## Discussion

The current study presents a new molecular mechanism regulating the insulin-sensitising effect of acute RE on skeletal muscle. Here, we provided evidence that a single bout of acute RE increases insulin-stimulated skeletal muscle glucose uptake, and this insulin-sensitising effect is augmented by inhibition of rapamycin-sensitive mTORC1 activation and subsequent IRS-1 Ser632/635 and Ser612 phosphorylation.

IRS-1 Ser phosphorylation is one of the mechanisms inducing insulin resistance in various tissues of patients and rodents with type 2 diabetes^47–51^. Morino *et al*.^52^ suggested that reduction of insulin-stimulated Akt phosphorylation was associated with increased IRS-1 phosphorylation at human Ser (hSer) 312, hSer616, and hSer636 (equivalent to Ser307, Ser612 and Ser632 in rat IRS-1, respectively) in skeletal muscle of insulin-resistant humans. Bouzakri *et al*.^48^ and Bandyopadhyay *et al*.^47^ also showed increases in IRS-1 hSer636 (rat: Ser632) and hSer307 (rat: Ser302) in patients with type 2 diabetes. Moreover, increased Ser phosphorylations or mutation on the sites in IRS-1 increase or decrease insulin resistance, respectively (i.e., hSer270/rat Ser265, hSer666/rat Ser662, hSer794/rat Ser789 and hSer1101/rat Ser1100)^53–56^. Accordingly, increasing IRS-1 Ser phosphorylation should impair insulin sensitivity. Some mechanisms increasing IRS-1 Ser phosphorylation are explained by increases in mTORC1 activation^42,44,49,53,54^. Gual *et al*.^44^ reported that Akt signal activation caused increases in IRS-1 phosphorylation at Ser302, Ser612 and Ser632 (rat phosphorylation sites) that were blocked by rapamycin in skeletal muscle and adipose tissue. Tremblay *et al*.^53^ and Khamzina *et al*.^49^ also showed that obesity-induced increases in mTORC1 activation and IRS-1 hSer1101 (rat: Ser1100) and hSer636/639 (rat: Ser632/635) phosphorylation were inhibited by rapamycin in skeletal muscle, adipose or hepatic tissue (or cell). In the present study, acute RE activated mTORC1 and increased phosphorylation of IRS-1 Ser612, Ser632/635 and Ser1100, all of which, except for IRS-1 Ser1100, were blocked by rapamycin. Following these responses, insulin-stimulated Akt signal activation after RE was impaired, but not in rapamycin-treated rats. Moreover, acute RE activated mTORC1 to a greater extent and for a longer duration than did AE in humans^34,35^. Taken together, these data suggest that acute RE-induced prolong mTORC1 activation and subsequent IRS-1 Ser612 and 632/635 phosphorylation attenuates acute exercise-induced insulin-sensitising effect in skeletal muscle.

In principle, rapamycin inhibits rapamycin-sensitive mTORC1 pathway activation in response to acute RE in both humans and rodents^36,57^. IRS-1 phosphorylations at Ser307, Ser612, Ser632/635, and Ser1100 were inhibited by rapamycin in skeletal muscle, adipose, and hepatic tissues and cells^44,49,53^. Accordingly, we showed that rapamycin inhibited mTORC1 activation and subsequent IRS-1 Ser612 and 632/635 phosphorylation after RE. However, RE-induced IRS-1 Ser1100 phosphorylation was not inhibited by rapamycin. Tremblay *et al*.^53^ suggested that the inhibition IRS-1 hSer1101 phosphorylation by rapamycin occurred under amino acid stimulating conditions, but not non-stimulating conditions. Furthermore, protein kinase C θ was shown to be an upstream regulator of IRS-1 hSer1101 (rat: Ser1100)^58^. These data suggest that RE might increase IRS-1 Ser1100 phosphorylation independent of mTORC1 activation.

Rapamycin is well-known as a highly selective inhibitor of mTORC1^45^. Therefore, rapamycin was widely used to identify the role of mTORC1. Bentzinger *et al*.^59^ have generated skeletal muscle-specific the mTORC1 component raptor knockout mouse; however, the mice have exhibited muscular dystrophy^59^ and developed insulin resistance^60^. These phenotype affects exercise quality and post-exercise insulin sensitivity, although rapamycin did not change the total workload of RE (data are not shown) and insulin-stimulated muscle glucose uptake in both previous and present studies^61^. Therefore, the conventional raptor knockout model mouse was not the best method to determine the role of mTORC1 on insulin sensitivity after acute RE. In 2019, tamoxifen-inducible raptor knockout mice were newly generated, and it minimised chronic adaptation by raptor deletion^62^. Thus, in the future study, we can explain the specific role of mTORC1 on insulin-sensitising effect following RE by using the inducible raptor knockout model mice.

In previous studies, an increase in skeletal muscle insulin sensitivity after AE was not associated with enhanced proximal insulin signaling in humans and rodents^3,8,9^. Furthermore, insulin-stimulated IRS-1 Tyr phosphorylation did not differ between the rested and the exercised legs, although the glucose uptake of the exercised leg during insulin clamp was enhanced^9^. By contrast, the increase in insulin-stimulated skeletal muscle glucose uptake after AE was diminished by skeletal muscle-specific AMPKα1/α2 knockout mice^15^. Therefore, they suggested that AMPK activation, but not IRS-1/Akt signaling in response to exercise, was important for muscle insulin sensitivity. However, in the case of RE, we found that prolonged mTORC1/IRS-1 signal activation by exercise may attenuate insulin-stimulated Akt/TBC1D4 signal activation, although AMPK was activated immediately. These results suggest that exercise mode, causing elevated and prolonged mTORC1 activation, interrupts prior AMPK activation–related increases in insulin sensitivity in skeletal muscle.

Theoretically, skeletal muscle glucose uptake should reflect Akt signal activation. However, insulin-stimulated glucose uptake was improved by RE, although Akt signal was impaired in this state (Fig. 5). Interestingly, previous studies suggested that TBC1D4 phosphorylation at Thr649 and Ser711 (equivalent to Thr651 and Ser713 on rat, respectively) were important sites for the insulin-sensitising effect of prior AMPK activation^15–18^. Moreover, TBC1D4 Ser711 (rat; Ser713) was more reflective of increasing insulin sensitivity by prior exercise than was Thr649 (rat: Thr651)^63,64^. In the present study, we only measured TBC1D4 phosphorylation at Thr651; we found that the phosphorylation in response to insulin was impaired, as Akt, in the exercised leg. Although we did not measure Ser713, we could speculate that this phosphorylation under insulin stimulation might be lowered by impairment of Akt signaling. Nevertheless, a prior AMPK activation by RE might still have further facilitated insulin-stimulated Ser713 phosphorylation and glucose uptake. Therefore, we need to confirm this in a future study.

As the other limitation, based on the previous finding that skeletal muscle AMPKα1/α2 deletion diminished insulin-sensitising effect of *in-situ* muscle contraction and running exercise^15^, we expected the role of AMPK on RE-induced insulin-sensitising effects. If we could inhibit mTORC1 activation on skeletal muscle-specific AMPK knockout animals, we could directly identify whether AMPK knockout diminishes the enhanced insulin-sensitising effect of RE by inhibiting mTORC1. Additionally, we have not used a female rat for this present study because the menstrual cycle affects insulin sensitivity^65^. However, it is also important to show whether current evidence can replicate in female rats. Thus, the current evidence will be extended by an additional study confirming the role of mTORC1 on RE-induced insulin-sensitising effect in both male and female AMPK knockout animals.

Overall, we provided evidence that mTORC1 activation and subsequent IRS-1 Ser phosphorylation opposed the insulin-sensitising effect of acute RE on skeletal muscle. Although mTORC1 activation was thought to be the most important target for skeletal muscle hypertrophy by chronic resistance training^35–37^, our results newly suggested that mTORC1 activation could be a negative factor for acute RE mediating the increase in insulin sensitivity.

## Methods

### Ethical approvals

The study protocols were approved by the Ethics Committee for Animal Experiments at Ritsumeikan University (BKC2018-033). We do confirm that all experiments were performed in accordance with relevant guidelines and regulations.

### Animals

Male Sprague-Dawley (SD) rats, aged 10 weeks, were obtained from Japan SLC (Shizuoka, Japan). Animals were maintained at 22 °C–24 °C with 12-h light-dark cycles. Food (CE-2; CLEA Japan, Tokyo, Japan), and water were available *ad libitum*. After at least 1-week acclimatisation period, the animals were subjected to each experiment.

### Resistance-type exercise

Acute RE was mimicked as previously described^66^. Briefly, overnight-fasted rats were anaesthetised with isoflurane, and the right whole gastrocnemius muscle was subjected to maximal isometric contraction using percutaneous electrical stimulation (5 sets of 3-s stimulation × 10 contractions per set with 7-s intervals between contractions and 3-min rest between sets) with an electric stimulator and isolator (SS-104J; Nihon Kohden, Tokyo, Japan). The stimulation protocol called for 100 Hz, 4 ms and ~50 V. The left gastrocnemius muscle was saved as a non-exercise control. Muscle samples were obtained at either 0 or 6 h after RE. Six hours after RE, rats were assigned to the study identifying the effect of mTORC1 activation on insulin sensitivity, because the time point showed marked elevation of mTORC1 activity after the exercise^67,68^. Tissues were rapidly harvested and frozen in liquid nitrogen and stored at −80 °C until analysis. This RE method was established because stimulation induces 8–10% gastrocnemius muscle hypertrophy with 12–18 sessions in rats^67,68^.

### *In vivo* insulin stimulation

The exercised rats were anaesthetised with 2% isoflurane in air and were intraperitoneally injected with either insulin (2 U/kg body weight dissolved in saline; Novo Nordisk A/S, Bagsværd, Denmark) or saline 10 or 30 min before muscle sampling. This amount of insulin stimulation for 10 to 30 min was previously shown to increase skeletal muscle Akt pathway activation and decrease blood glucose levels in rats^69,70^.

### Inhibition of mTORC1 activity

Rapamycin was used for mTORC1 inhibition, as previously shown^57^. Briefly, rapamycin (1.5 mg/kg, 0.25 mg/mL in saline containing 0.5% dimethyl sulphoxide) or placebo (saline containing 0.5% dimethyl sulphoxide) was intraperitoneally injected 1 h before RE. Following the method of insulin stimulation, these rats were treated with insulin (2 U/kg body weight) at 5.5 h post-exercise, and then muscle samples were taken 30 min after insulin injection.

### Western blot analysis

Western blot analysis was performed as reported previously^68^. Briefly, frozen gastrocnemius muscles were powdered and homogenised in radioimmunoprecipitation assay buffer (Cell Signaling Technology, Danvers, MA, USA) supplemented with protease and phosphatase inhibitor cocktail (Roche Life Science, Indianapolis, IN, USA). Homogenates were centrifuged at 13,700 *g* for 20 min at 4 °C, and the protein concentrations of the supernatants were determined with a Protein Assay Rapid kit (Wako, Osaka, Japan). Equal volumes of lysates (2–20 μg) were separated by electrophoresis on 8%, 10%, or 12% sodium dodecyl sulphate–polyacrylamide gels, as appropriate. The proteins were subsequently transferred to PVDF membranes (Merck Millipore, Bedford, MA, USA) using a semidry method, and the membranes were washed in Tris-buffered saline containing 0.1% Tween 20 (TBST) and blocked with 5% powdered milk in TBST for 30 min at room temperature. The membranes were washed with TBST and incubated overnight with primary antibodies (1:1,000) against AMPKα phosphorylation (*p*-AMPKα)-Thr172 (Cat#2535), total AMPK (Cat#2793), *p*-ACC-Ser79 (Cat#11818), total ACC (Cat#3676), total TBD1D1 (Cat#5929), *p*-TBC1D4-Ser588 (Cat#8730) and *p*-TBC1D4-Thr642 (Cat#4288) (Rat: Ser597 and Thr651, respectively); total TBC1D4 (Cat#2670), *p*-P70S6K-Thr389 (Cat#9234), total P70S6K (Cat#2708), *p*-IRS-1-Ser1101(rat: Ser1100) (Cat#2385), Ser636/639 (rat: Ser632/635) (Cat#2388) and Ser612 (Cat#3203), total IRS-1 (Cat#3194), *p*-Akt-Thr308 (Cat#13038) and Thr473 (Cat#9271), and total Akt (Cat#4691) (Cell Signaling Technology, Danvers, MA, USA); and *p*-TBC1D1-Ser237 (rat: Ser231, Cat#07-2268) (Merck Millipore, Bedford, MA, USA). The probed membranes were washed in TBST and then incubated for 1 h at room temperature with the appropriate secondary antibodies (Cell Signaling Technology, Bedford, MA, USA). Immunoreactive bands were visualised by chemiluminescence (Luminata Forte Western HRP Substrate; Merck Millipore, Bedford, MA, USA) with an ImageQuant LAS 4000 imaging system (GE Healthcare, Amersham, UK). Densitometry was performed using ImageJ software version 1.46 (National Institutes of Health, Bethesda, MD, USA).

### *In vivo* 2-deoxy-d-glucose uptake

2-Deoxy-d-glucose (2DG) uptake method that we used was originally established by Saito *et al*.^71^ and widely used as *in-vivo* 2DG uptake measurement with some optimisations in previous studies, including ours^32,72,73^. Particularly, the anaesthetised rats were administered 2DG (166 nmol/g body weight) into a vein 20 min before muscle sampling. At the time of muscle sampling, gastrocnemius muscles were rapidly harvested, then frozen in liquid nitrogen. The frozen tissues were homogenised ultrasonically in 10 mmol/L Tris·HCl buffer (pH 8.1), heated at 95 °C for 15 min, and centrifuged at 17,800 *g* for 15 min at 4 °C. The transported 2DG into muscle accumulates as 2DG-6-phosphate (2DG6P); thus, the 2DG6P concentration in the supernatant was assessed with the enzyme cycling method (Nonradioactive 2DG Uptake Assay Kit; Cosmo Bio, Tokyo, Japan). In this method, we firstly oxidised glucose-6-phosphate (G6P) with a low concentration of glucose-6-phosphate dehydrogenase (G6PDH) and nicotinamide adenine dinucleotide (NAD^+^) to eliminate G6P in the lysate. As a second step, following the elimination of endogenous nicotinamide adenine dinucleotide phosphate (NADPH) and produced NADH, NADPH was produced through the oxidation of 2DG6P with a high concentration of G6PDH. The produced NADPH was used for quantification of 2DG6P with a microplate spectrophotometer (Bio-Rad, Hercules, CA, USA).

### Statistical analysis

Data are presented as means ± standard error. Two-way analysis of variance with repeated measures and paired/unpaired Students t-tests were used to assess statistical significance within and between interventions, where appropriate. *Post hoc* analysis was performed using t-tests with Benjamini-Hochberg false discovery rate correction, when appropriate. The main effects have been indicated by lines unless stated otherwise. Statistical significance was defined as P < 0.05.

## Supplementary information

Supplementary information.

## Data Availability

The datasets generated during and analysed during the current study are available from the corresponding author on reasonable request.
