# Supplementary material for: Enhanced skeletal muscle insulin sensitivity after acute resistance-type exercise is upregulated by rapamycin-sensitive mTOR complex 1 inhibition
Source: Sci Rep. 2020 May 22;10:8509. doi: 10.1038/s41598-020-65397-z (PMC7244536; doi:10.1038/s41598-020-65397-z)

Enhanced skeletal muscle insulin sensitivity after acute resistance-type exercise is upregulated by rapamycin-sensitive mTOR complex 1 inhibition

Kohei Kido, Kohei Sase, Takumi Yokokawa, Satoshi Fujita

Raw blots related to Figure 1

P-AMPKα Thr172

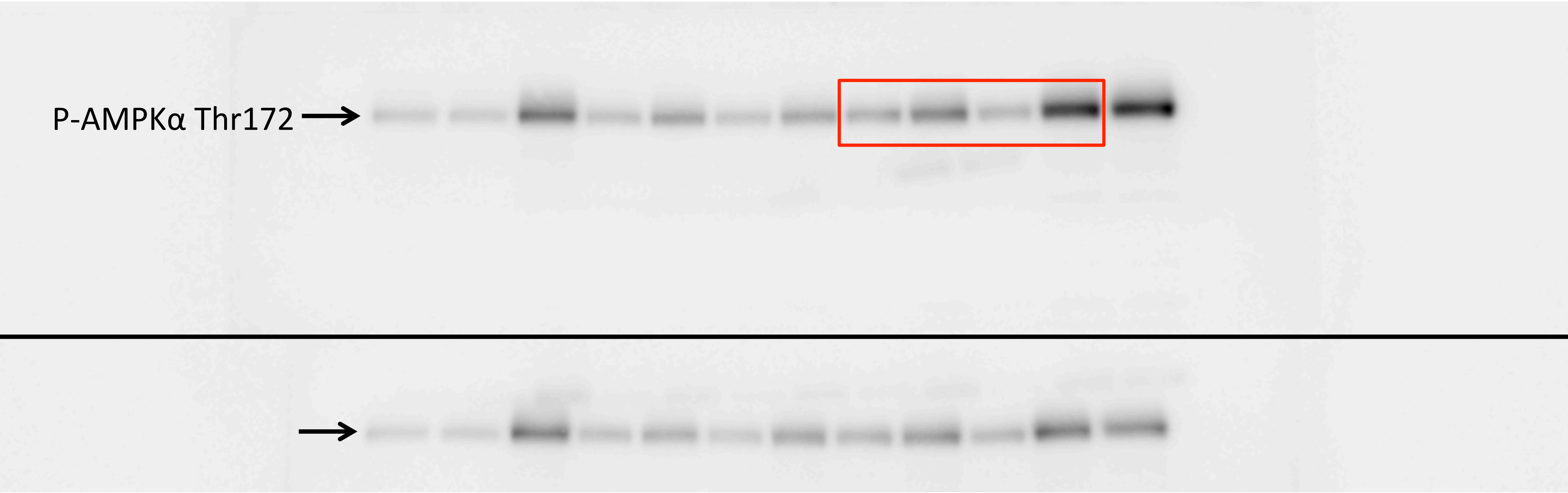

T-AMPK

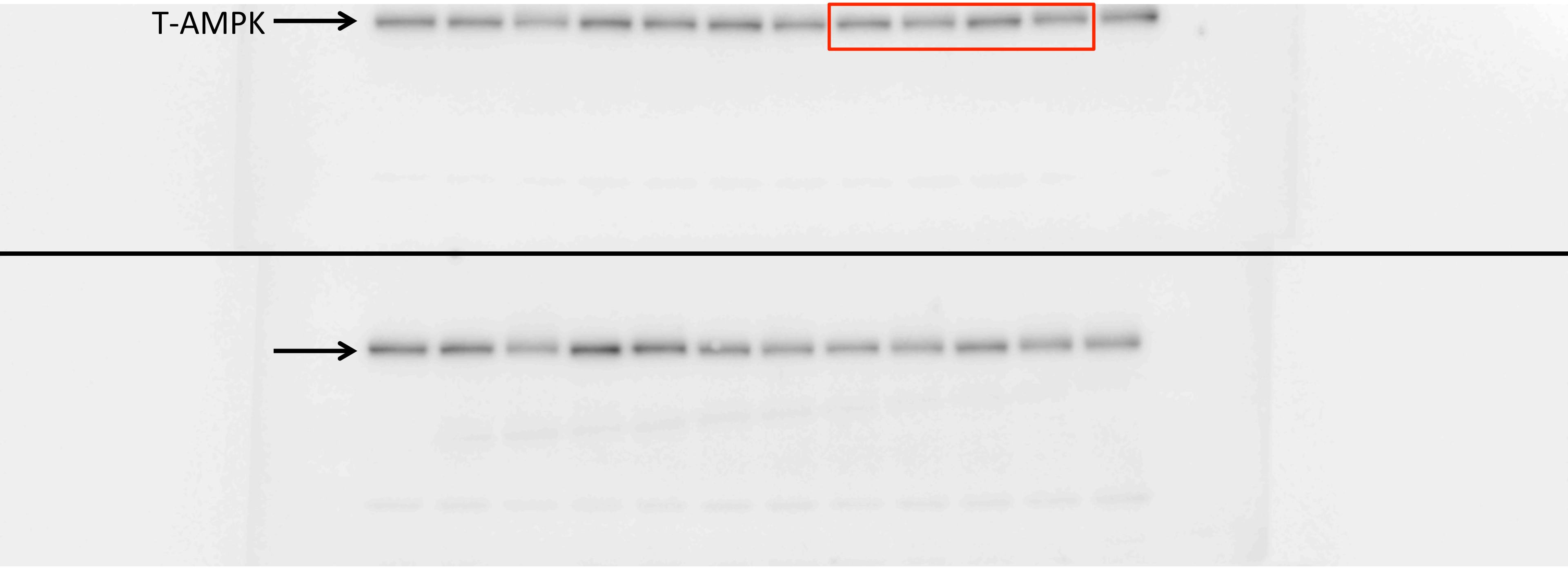

P-ACC Ser79

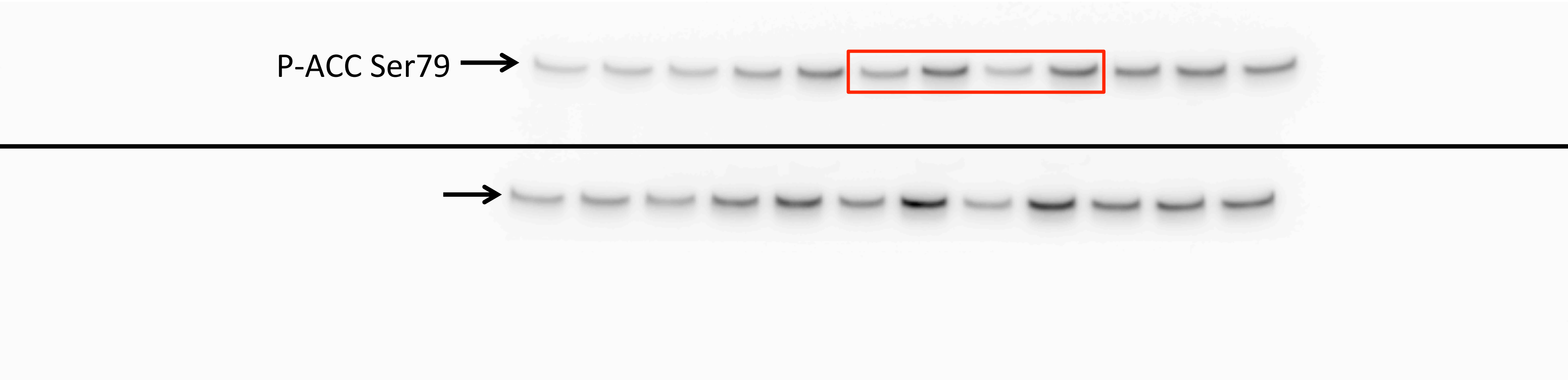

T-ACC

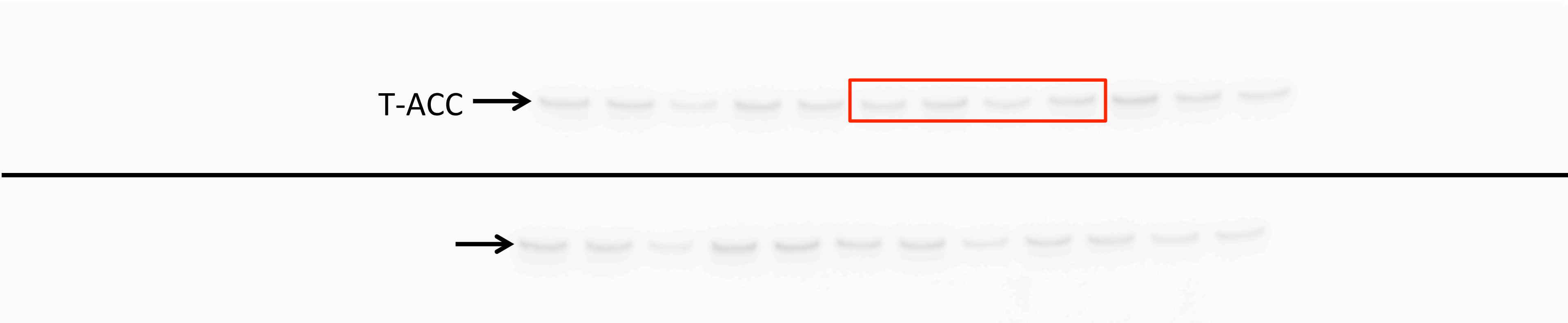

P-TBC1D1 Ser231
T-TBC1D4
P-TBC1D4 Thr651

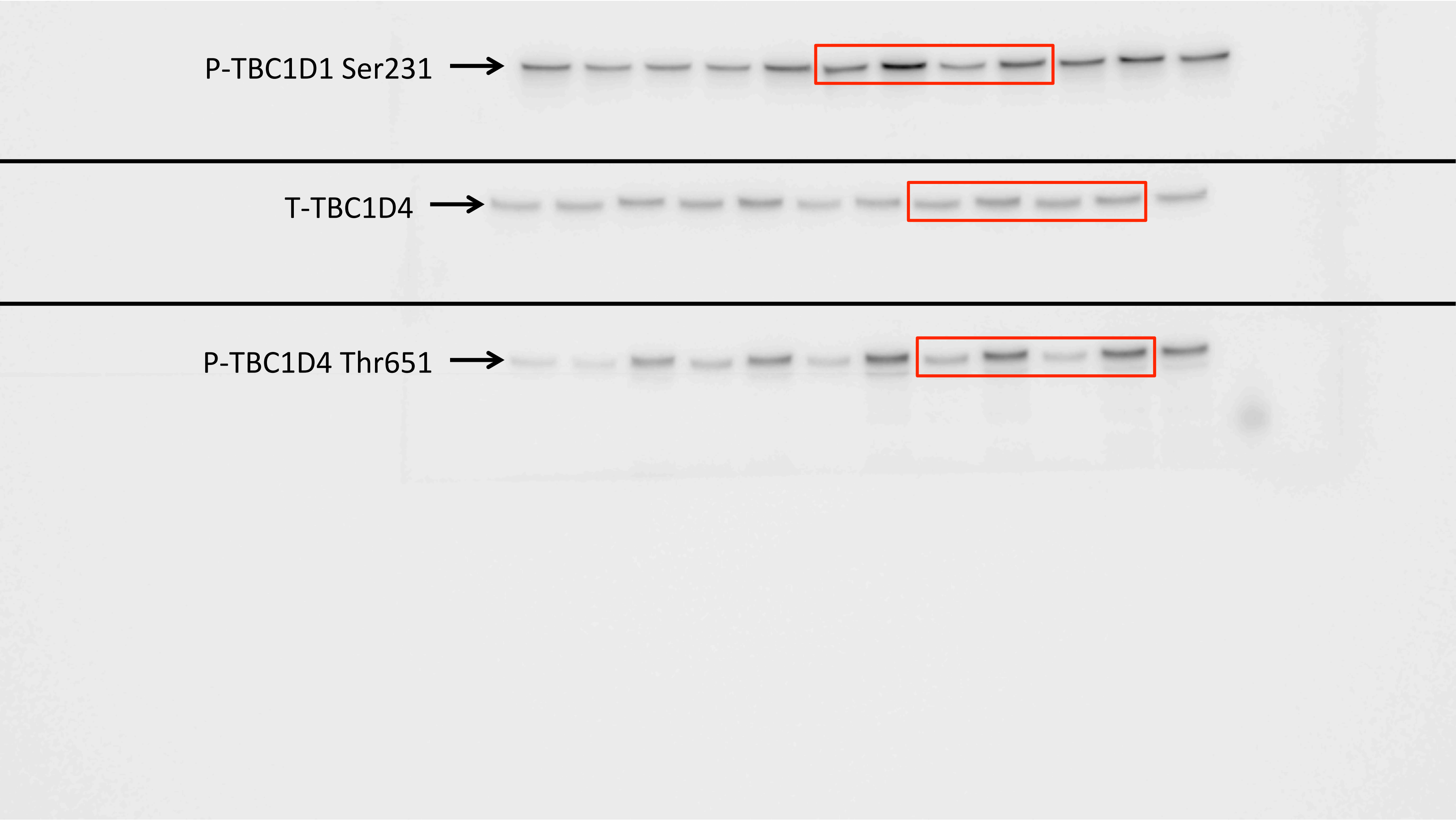

T-TBC1D1

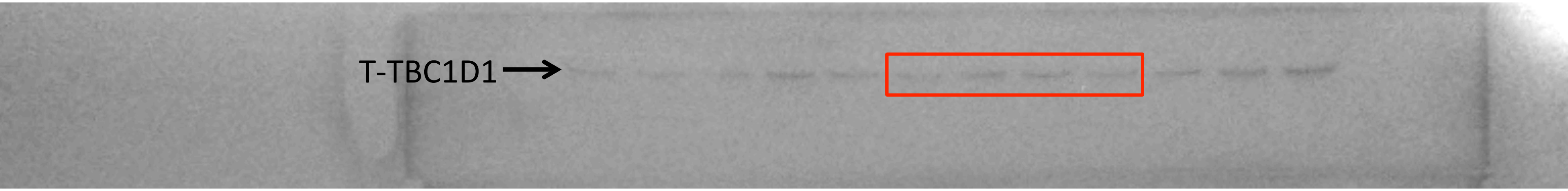

P-TBC1D4 Ser597

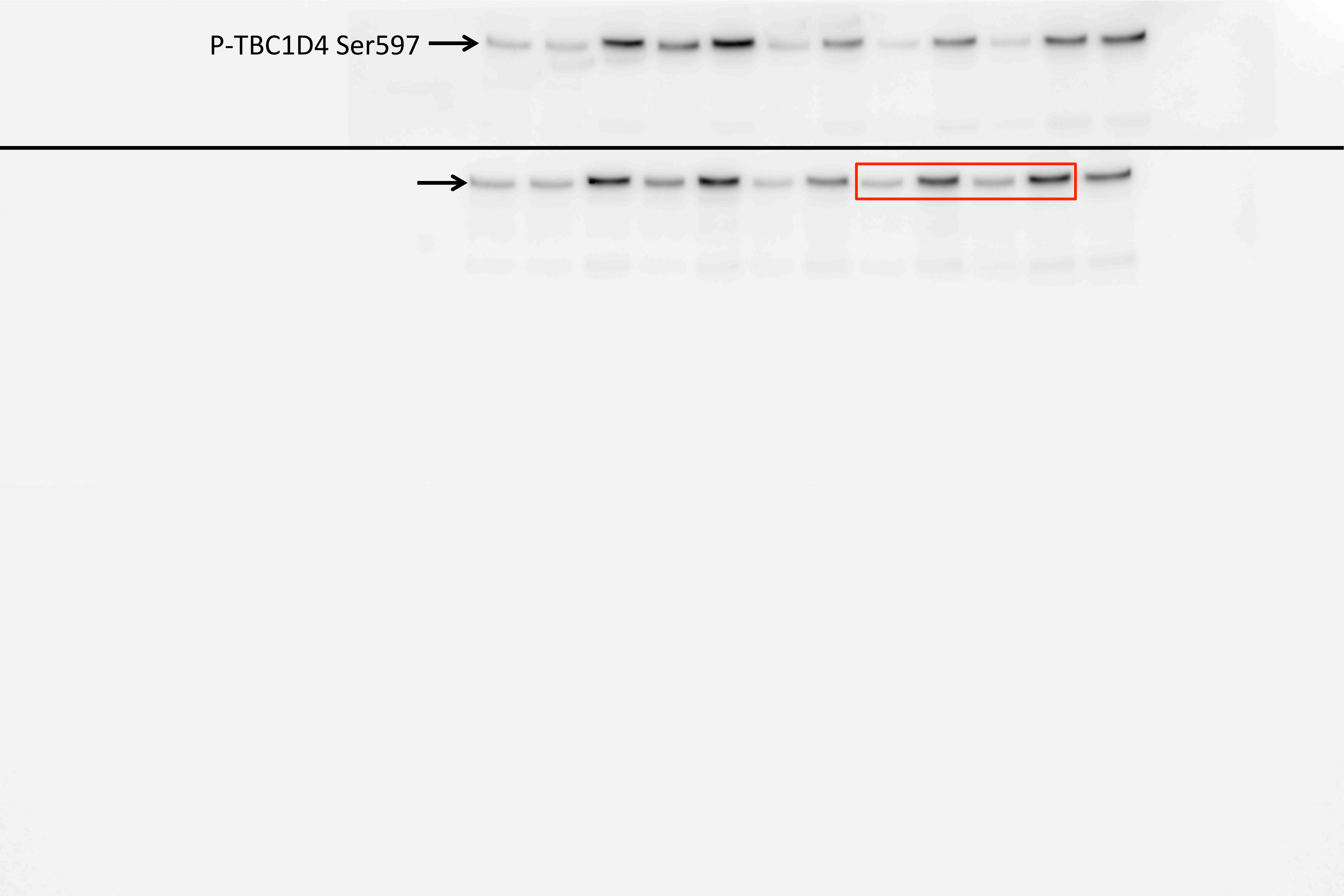

Enhanced skeletal muscle insulin sensitivity after acute resistance-type exercise is upregulated by rapamycin-sensitive mTOR complex 1 inhibition

Kohei Kido, Kohei Sase, Takumi Yokokawa, Satoshi Fujita

Raw blots related to Figure 2

P-P70S6K Thr389

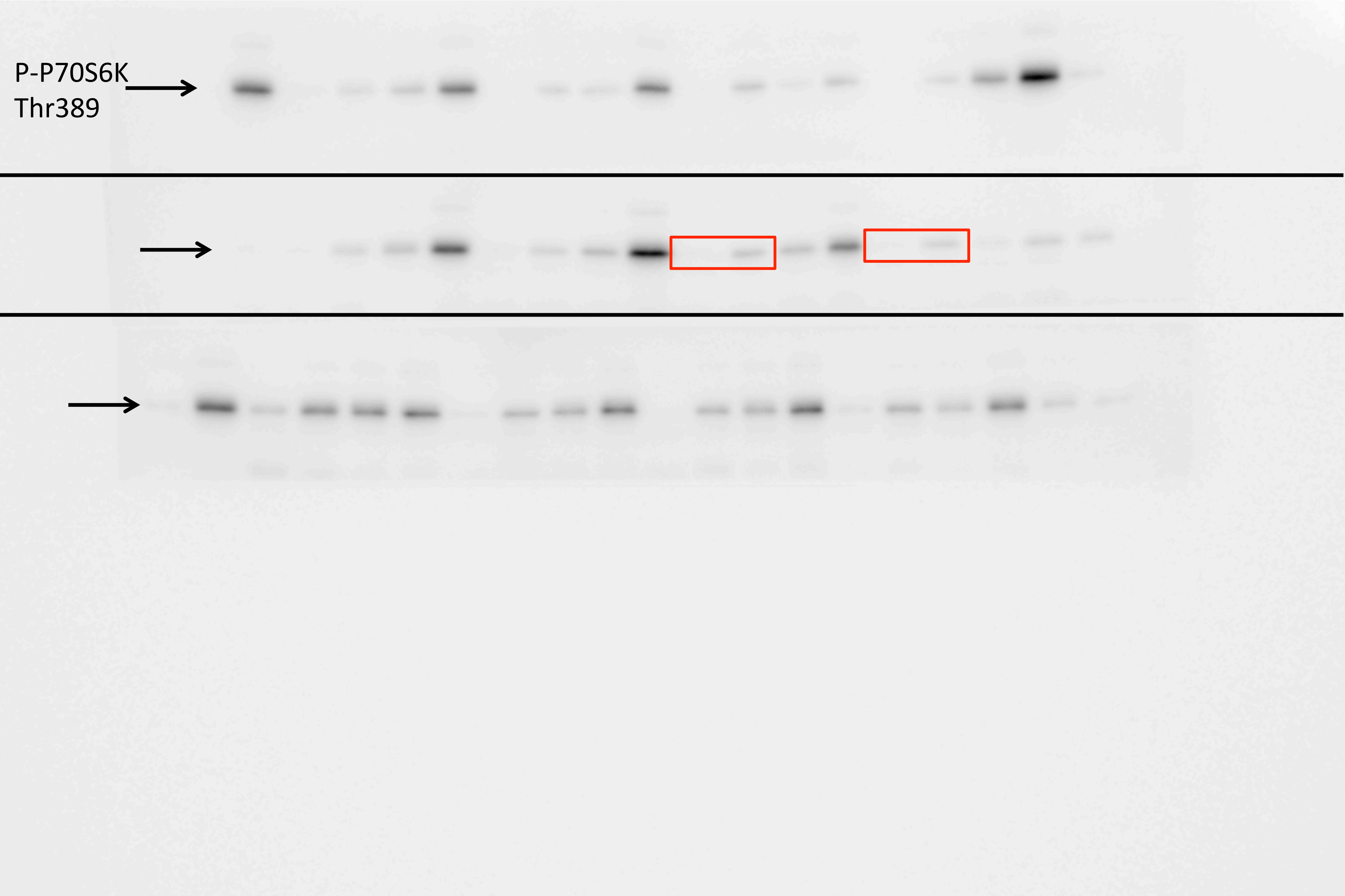

T-P70S6K

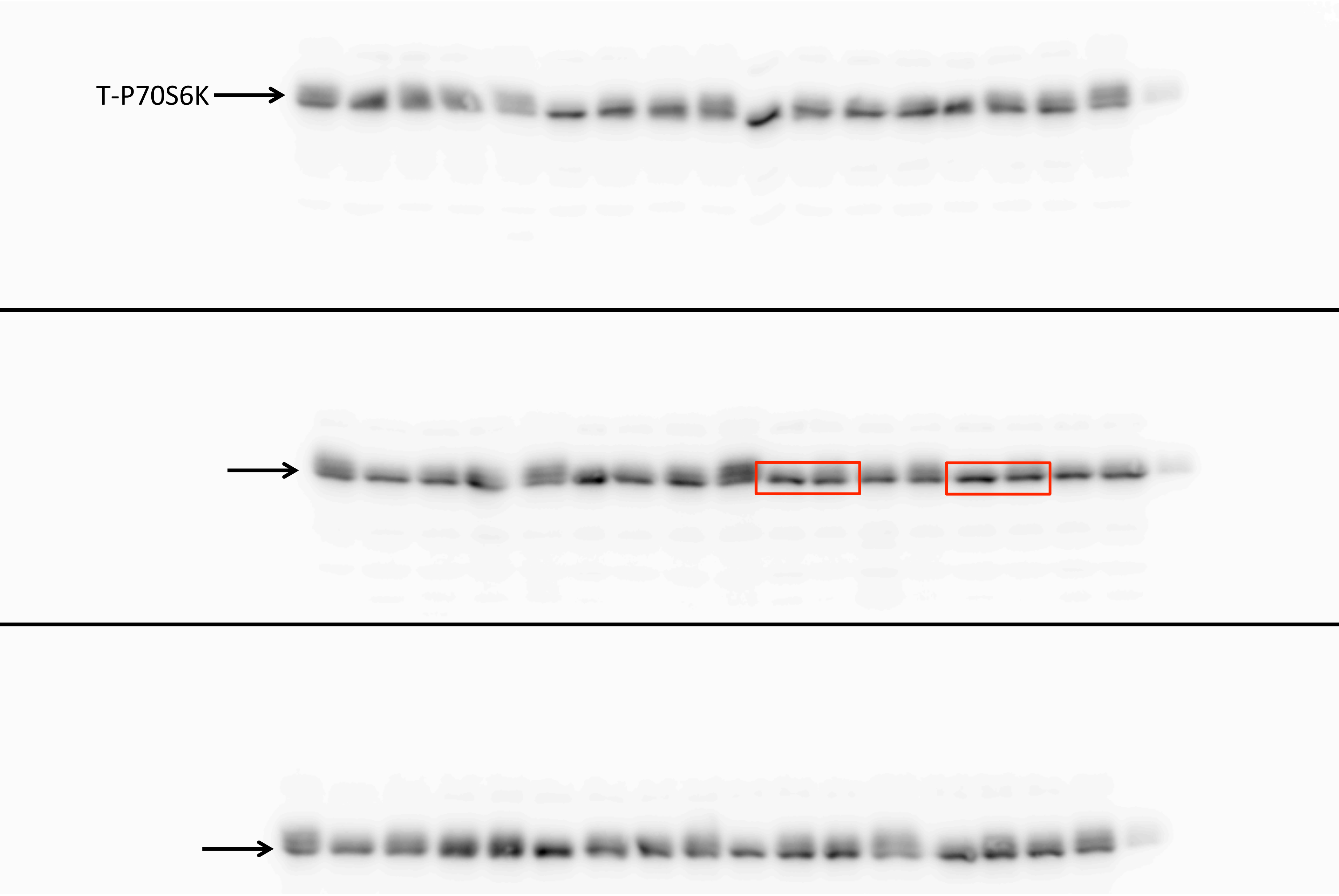

P-IRS-1 Ser1100

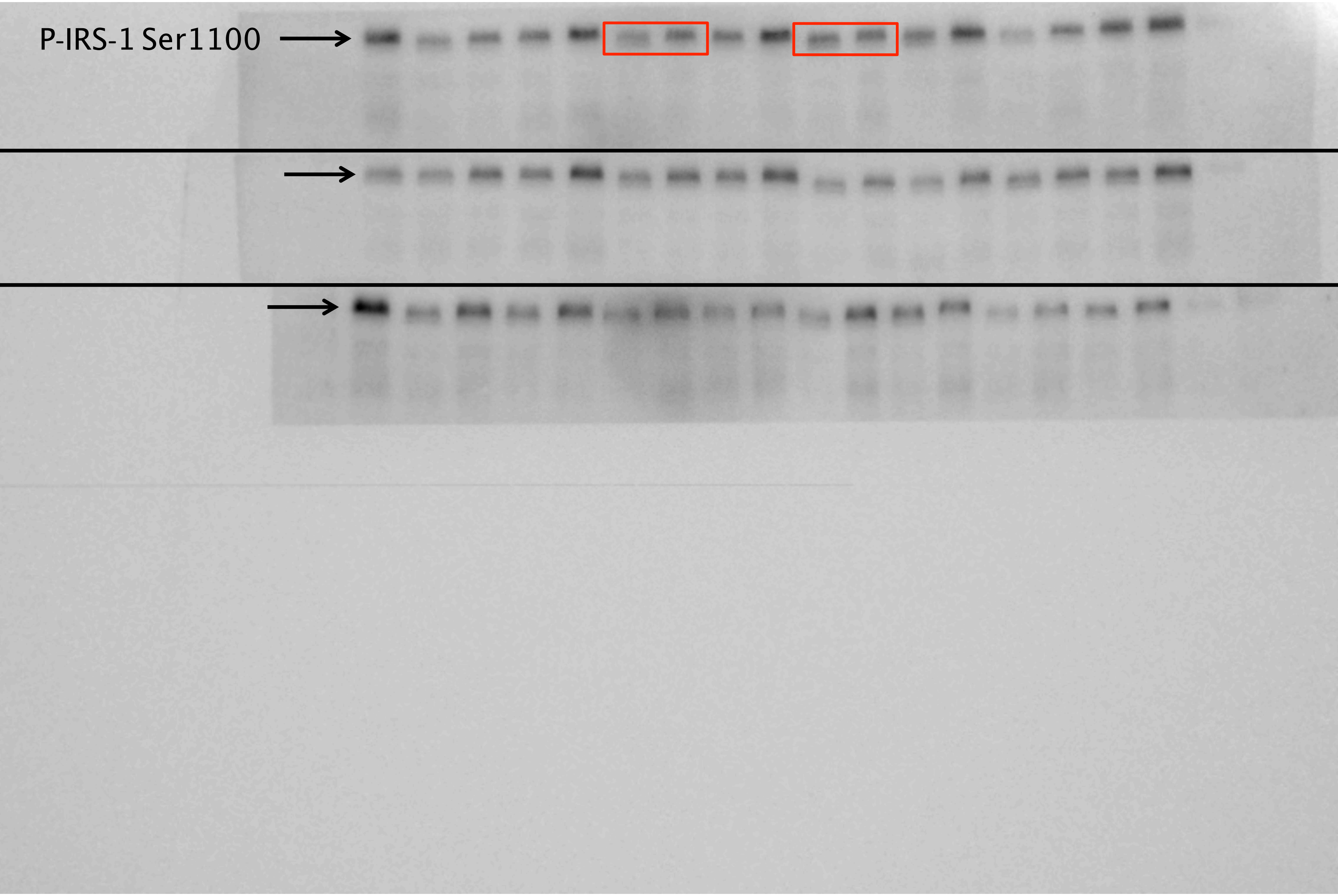

P-IRS-1 Ser632/635

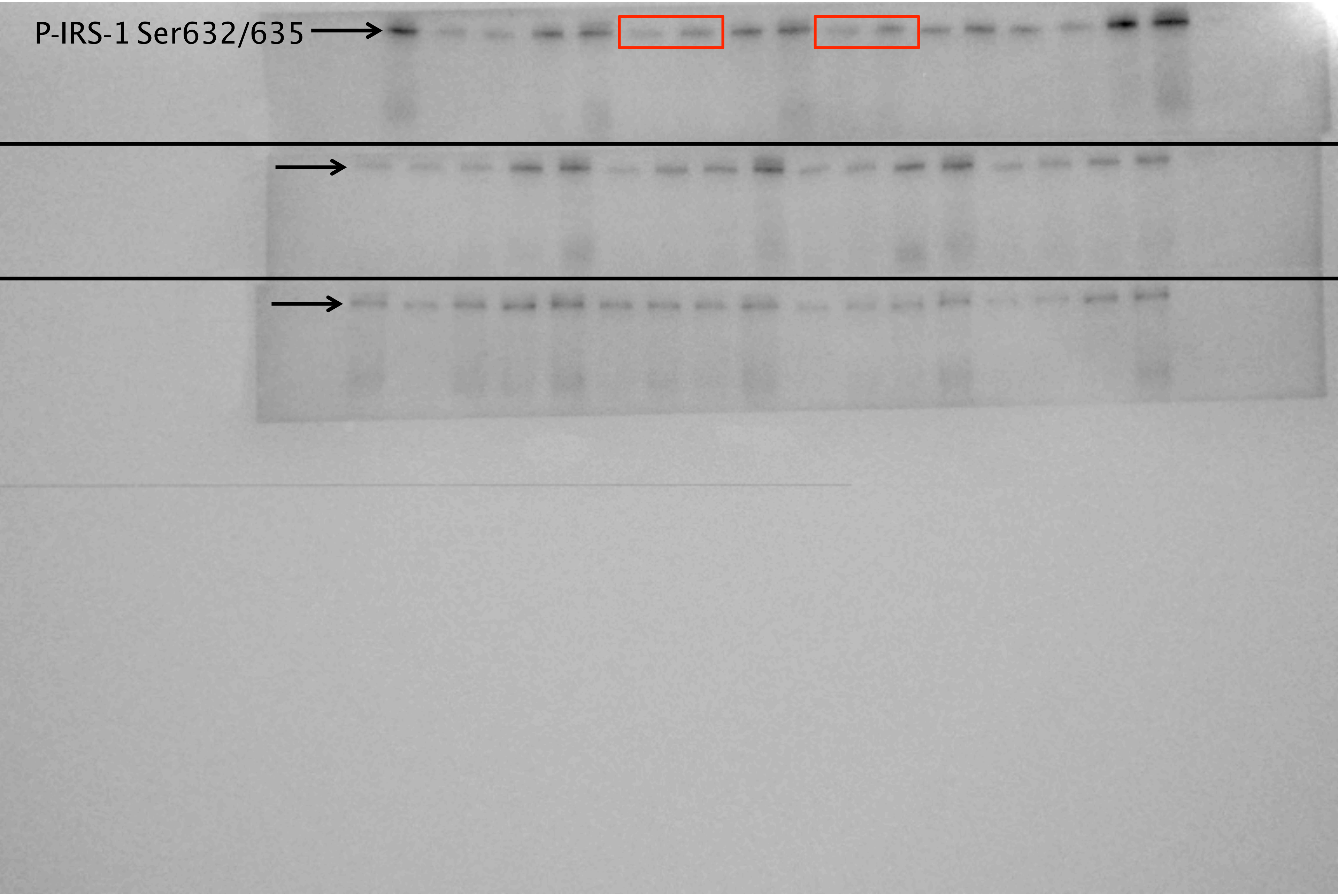

P-IRS-1 Ser612

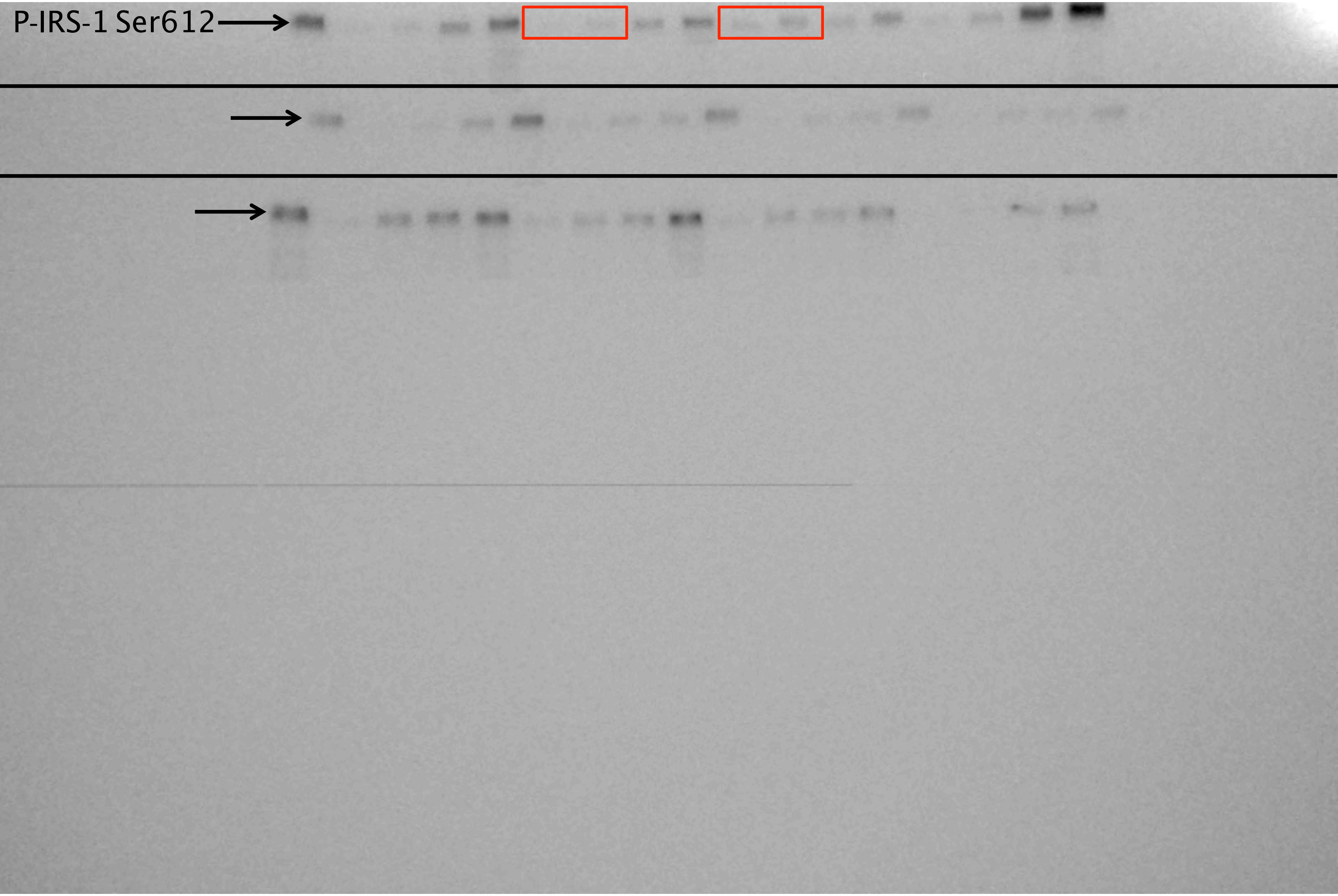

T-IRS-1

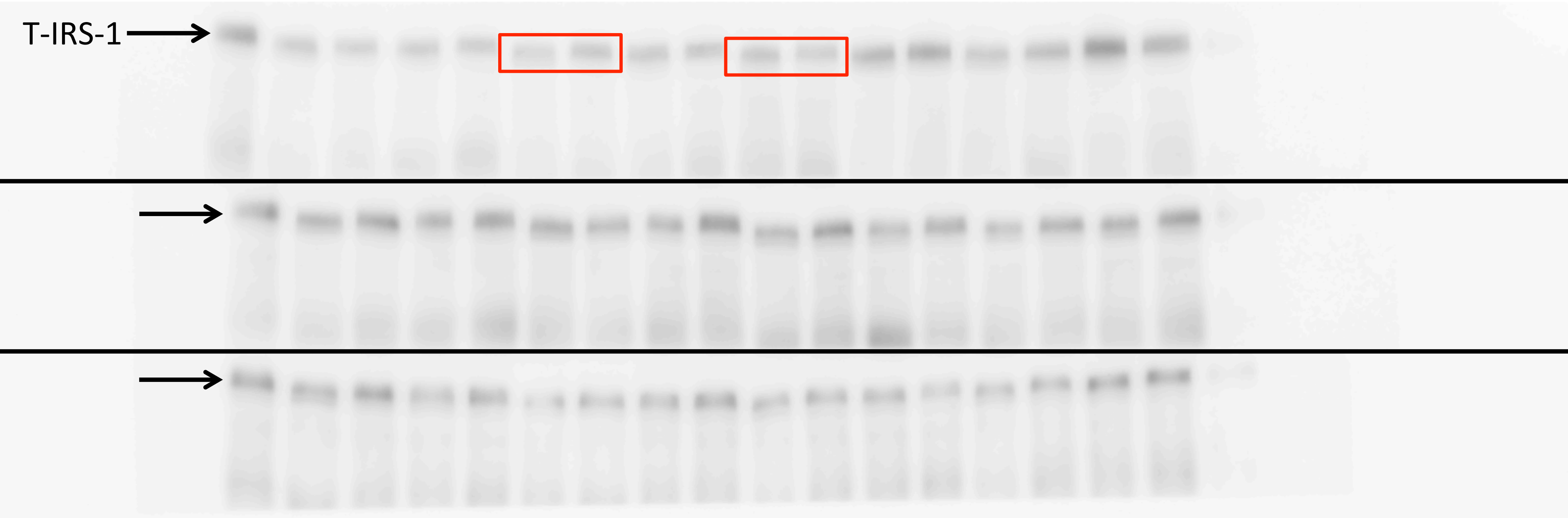

Enhanced skeletal muscle insulin sensitivity after acute resistance-type exercise is upregulated by rapamycin-sensitive mTOR complex 1 inhibition

Kohei Kido, Kohei Sase, Takumi Yokokawa, Satoshi Fujita

Raw blots related to Figure 3

P-Akt Thr308

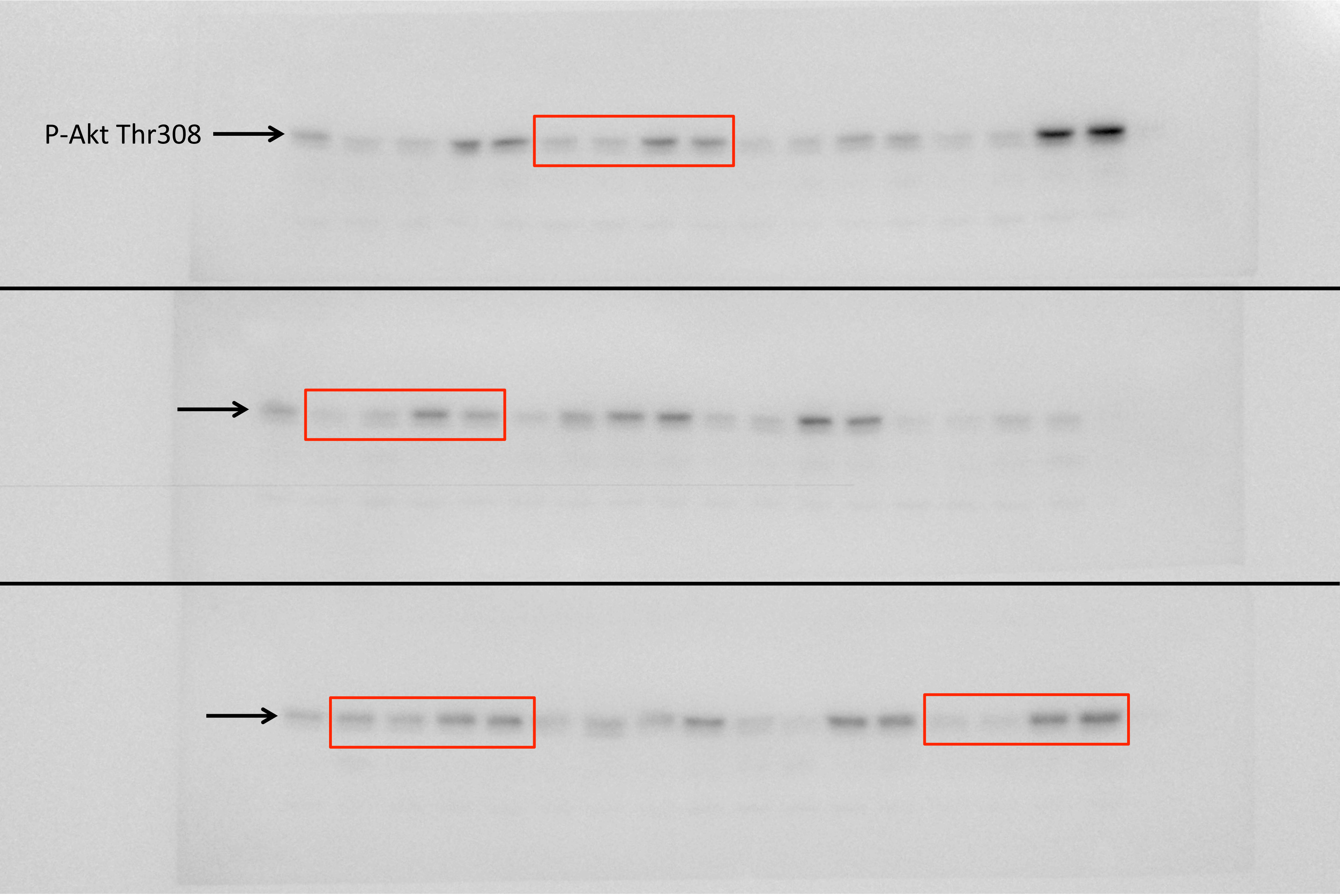

P-Akt Ser473

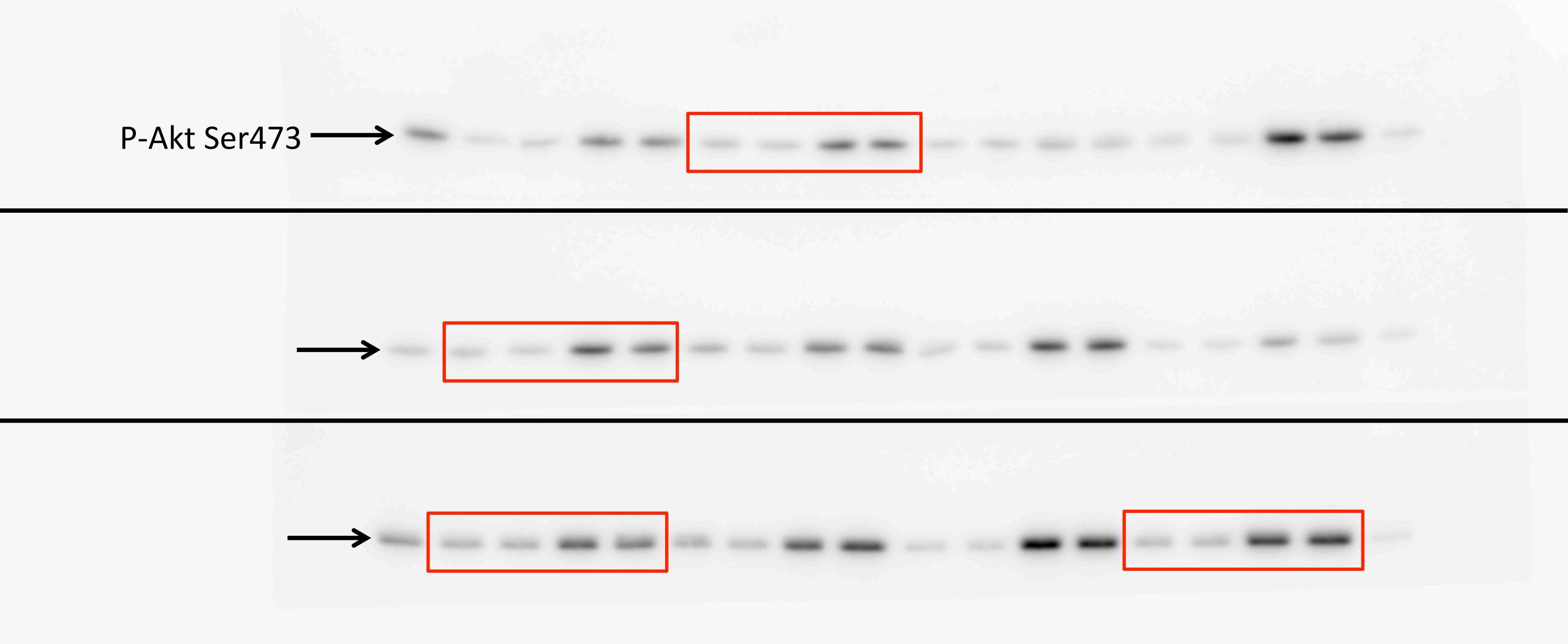

T-Akt

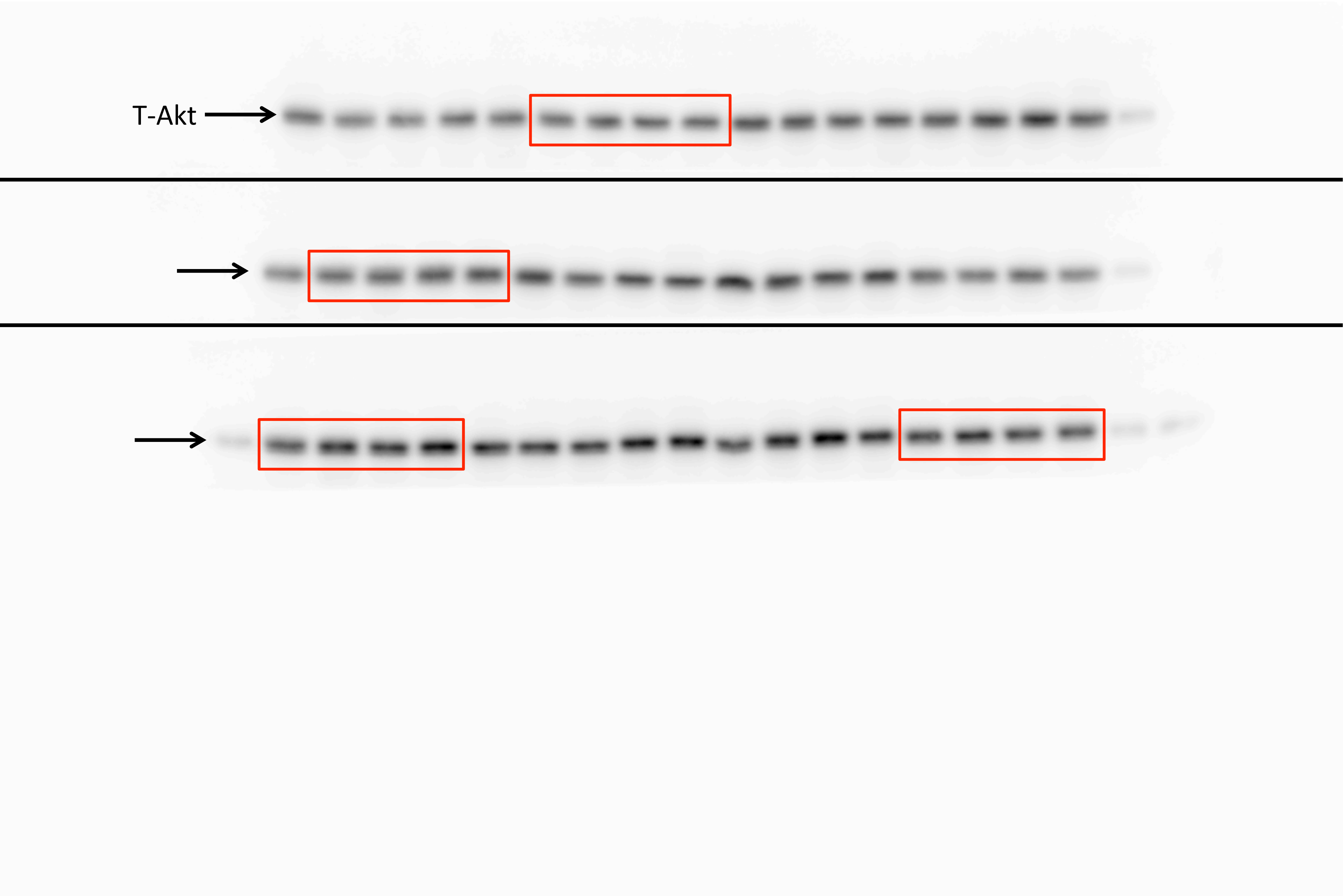

Enhanced skeletal muscle insulin sensitivity after acute resistance-type exercise is upregulated by rapamycin-sensitive mTOR complex 1 inhibition

Kohei Kido, Kohei Sase, Takumi Yokokawa, Satoshi Fujita

Raw blots related to Figure 4

P-TBC1D1 Ser231

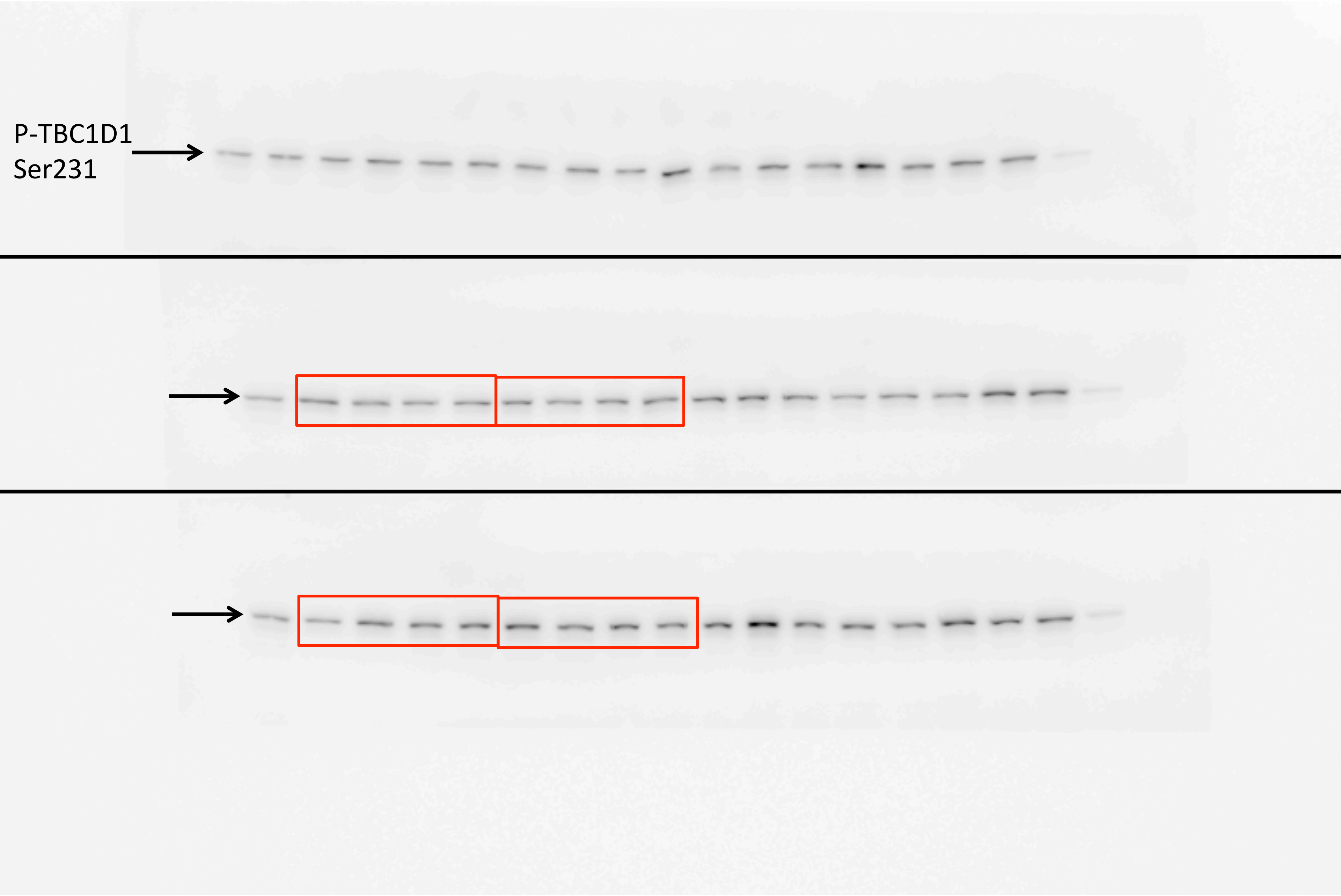

T-TBC1D1

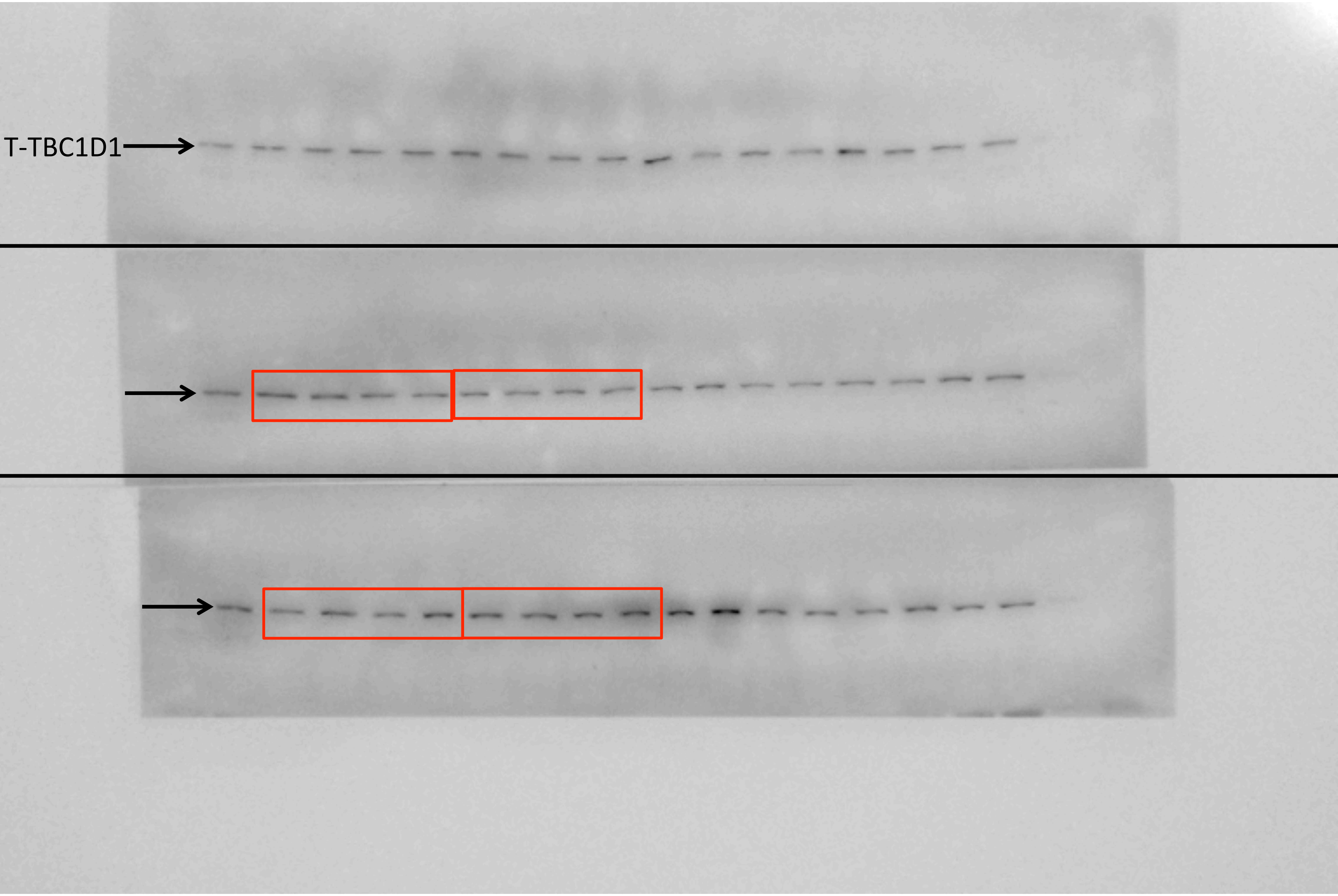

P-TBC1D4 Ser597

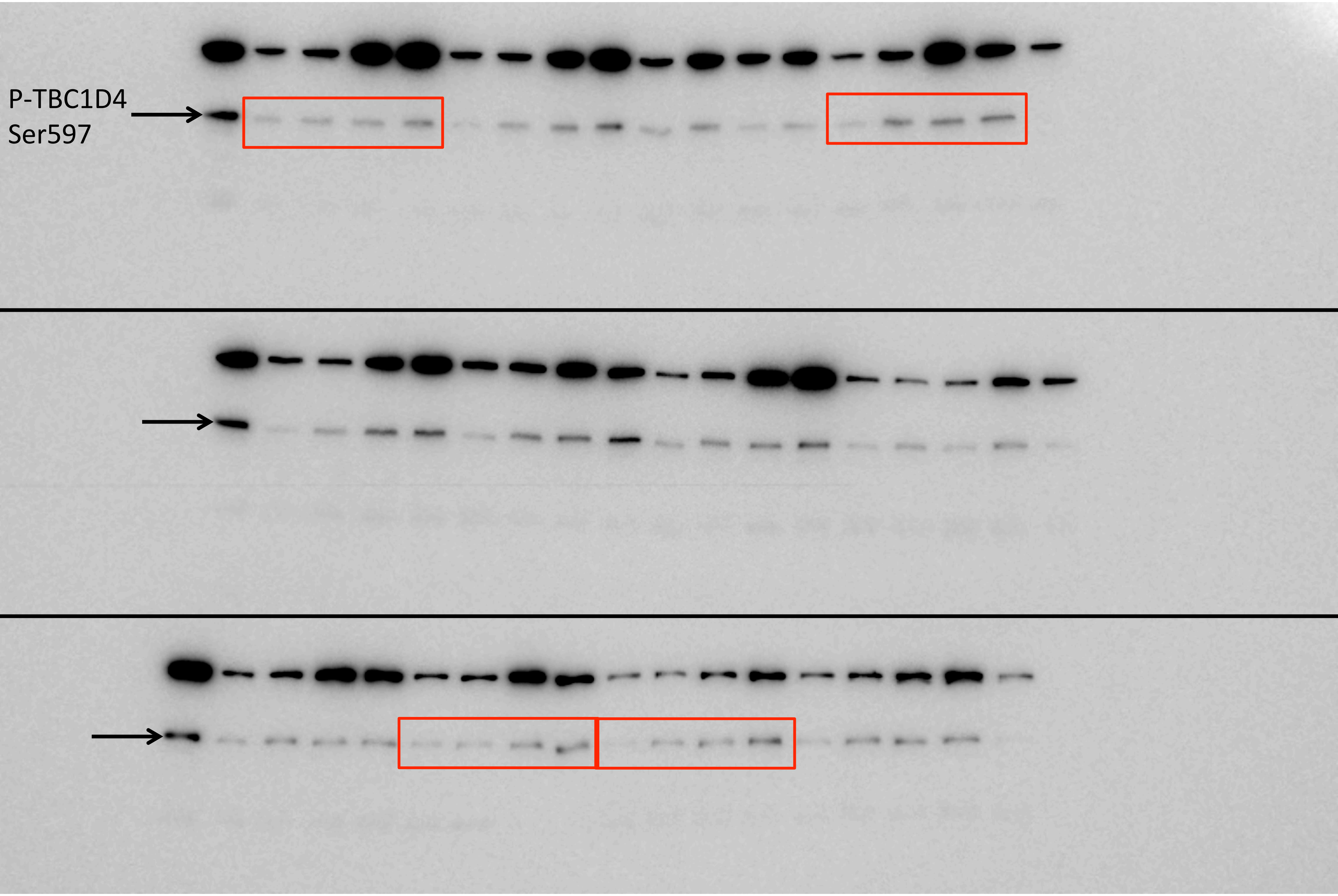

P-TBC1D4 Thr651

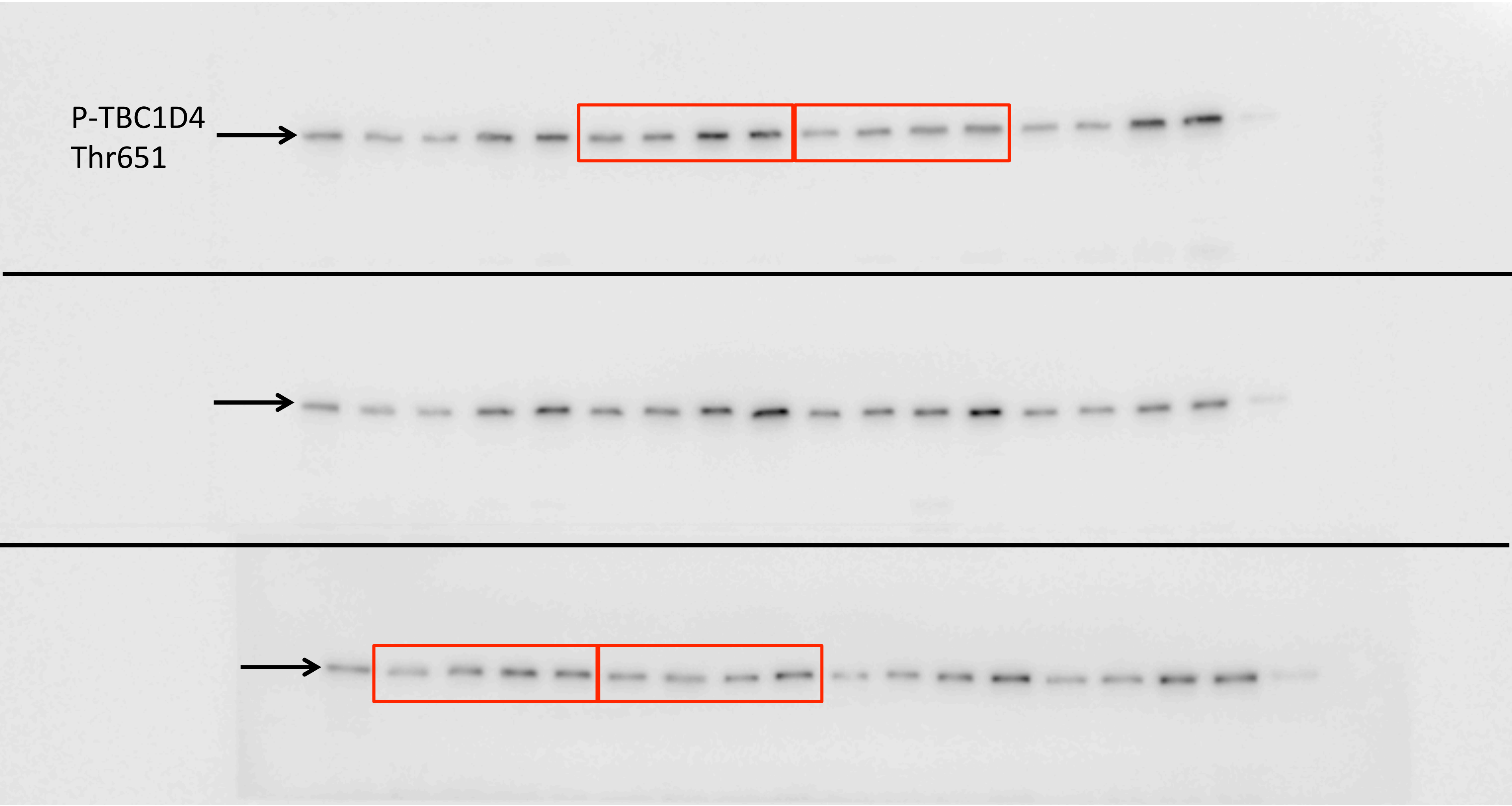

T-TBC1D4

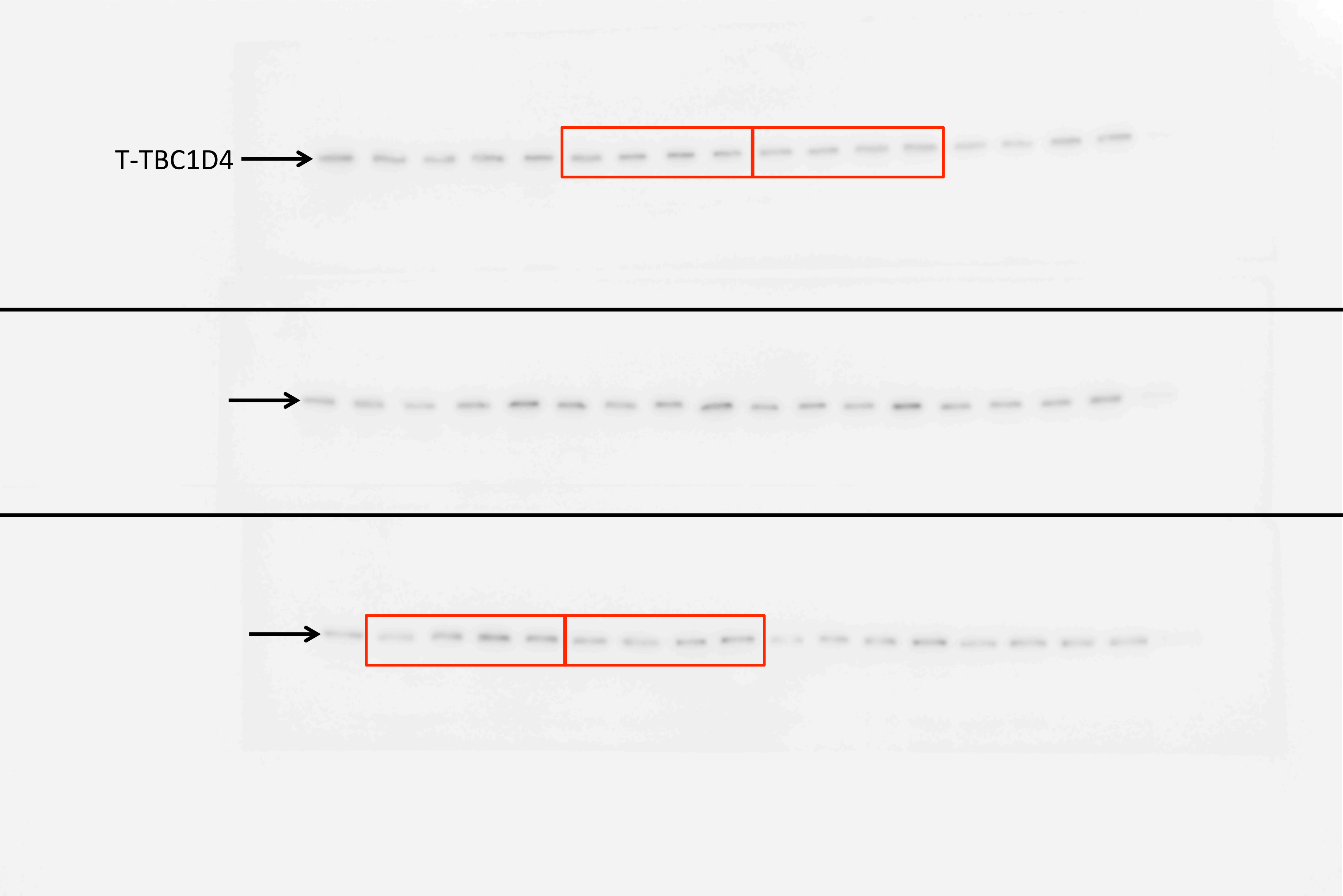

Enhanced skeletal muscle insulin sensitivity after acute resistance-type exercise is upregulated by rapamycin-sensitive mTOR complex 1 inhibition

Kohei Kido, Kohei Sase, Takumi Yokokawa, Satoshi Fujita

Raw blots related to Figure 6

P-P70S6K Thr389

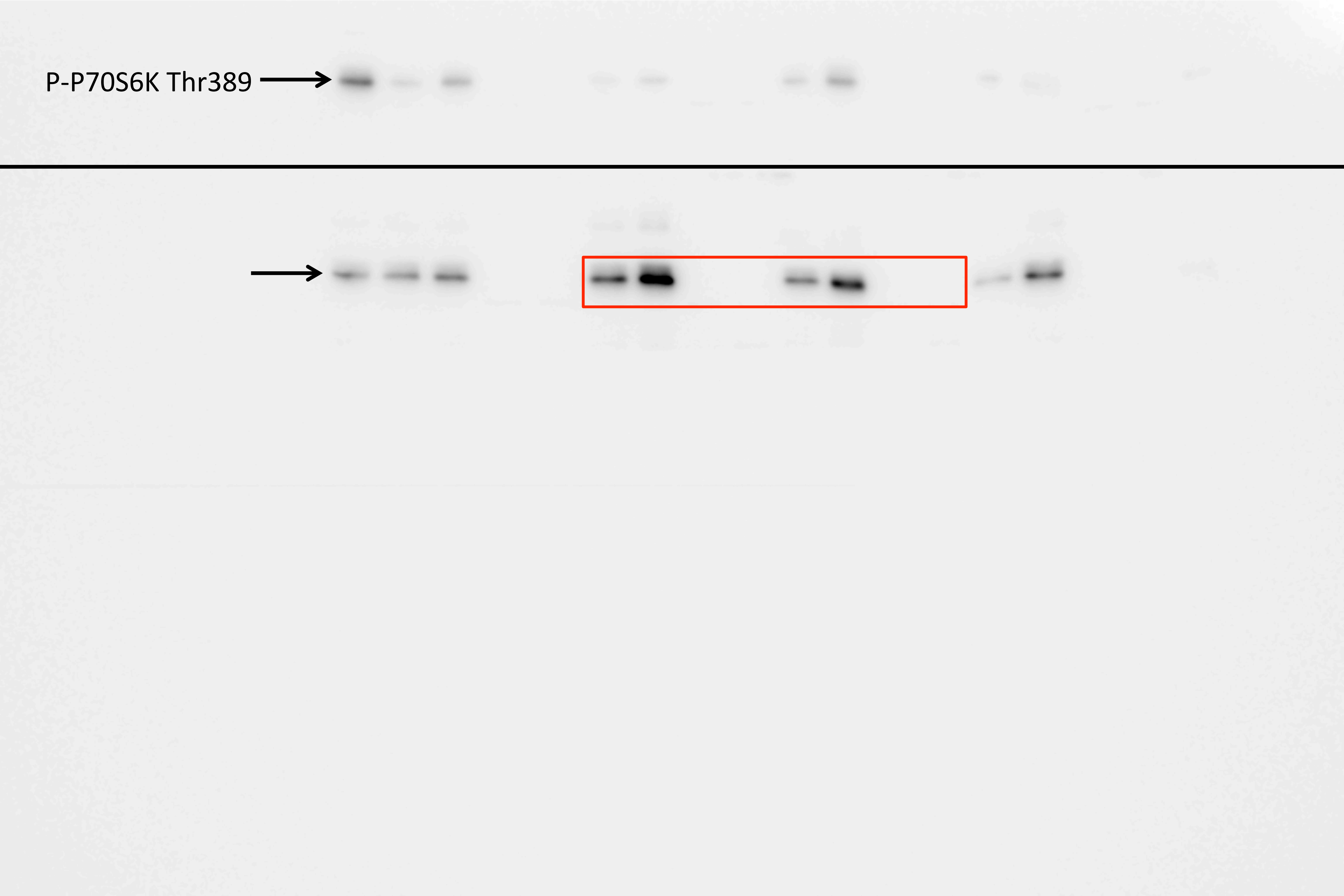

T-P70S6K

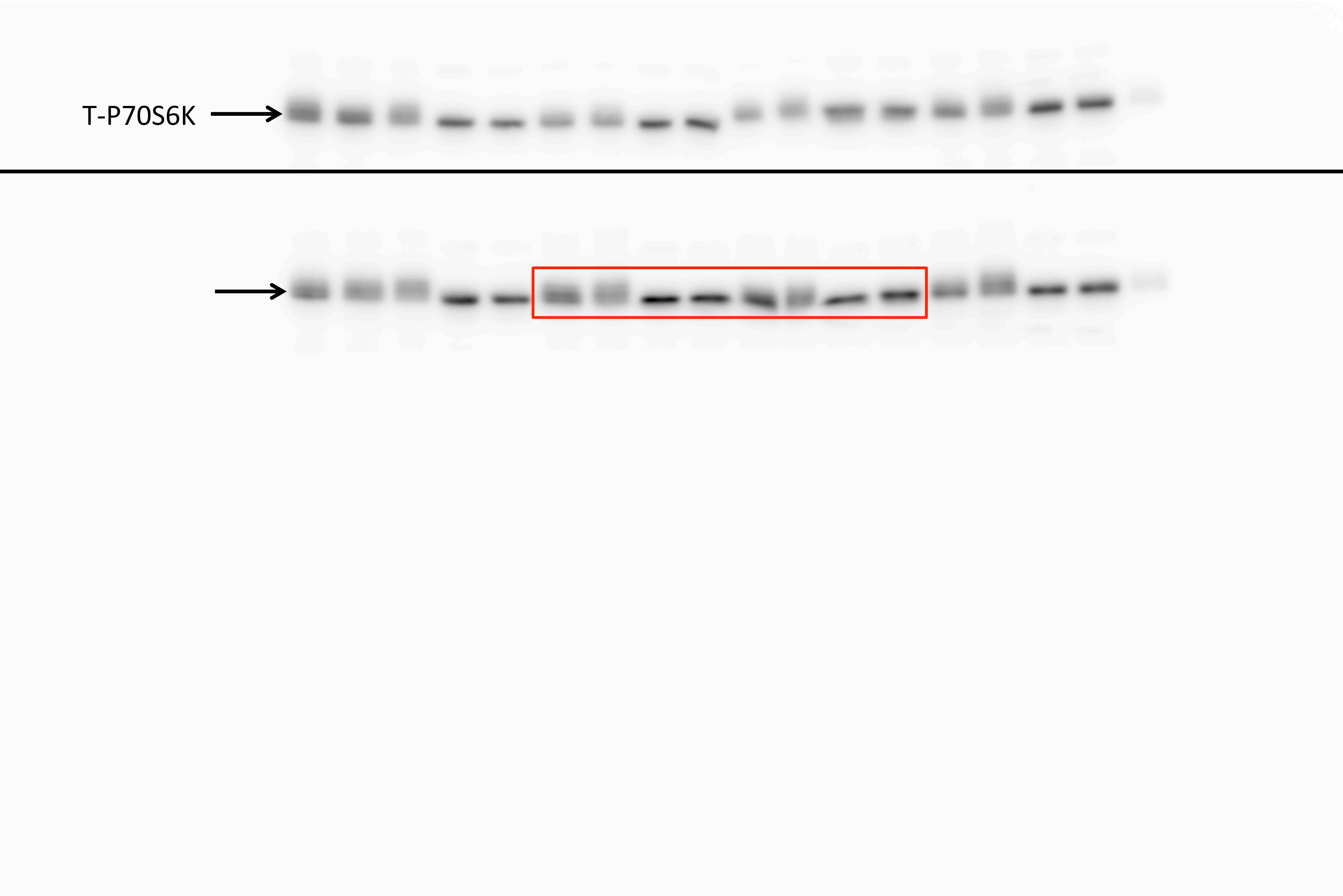

P-IRS-1 Ser1100

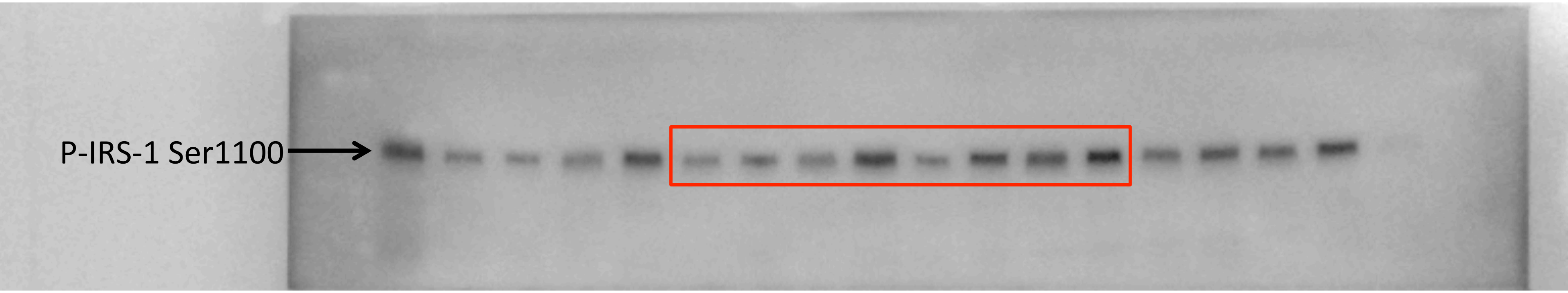

P-IRS-1 Ser632/635

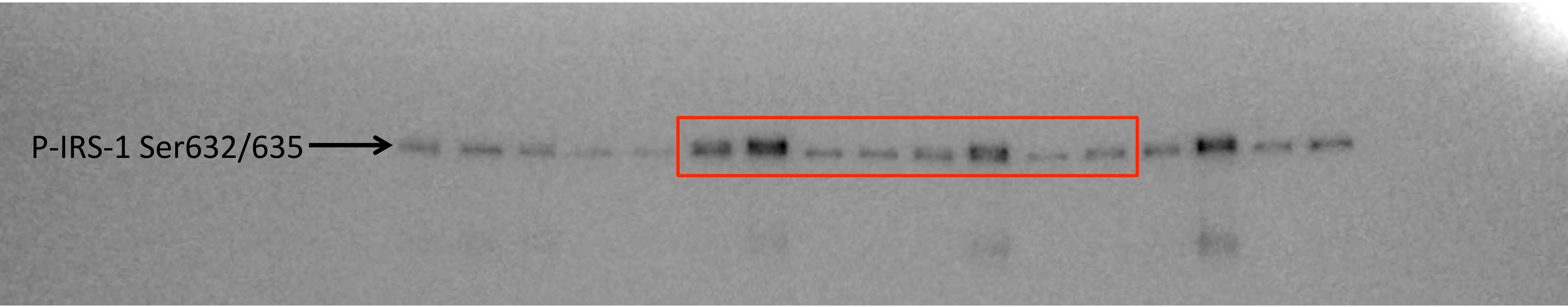

P-IRS-1 Ser612

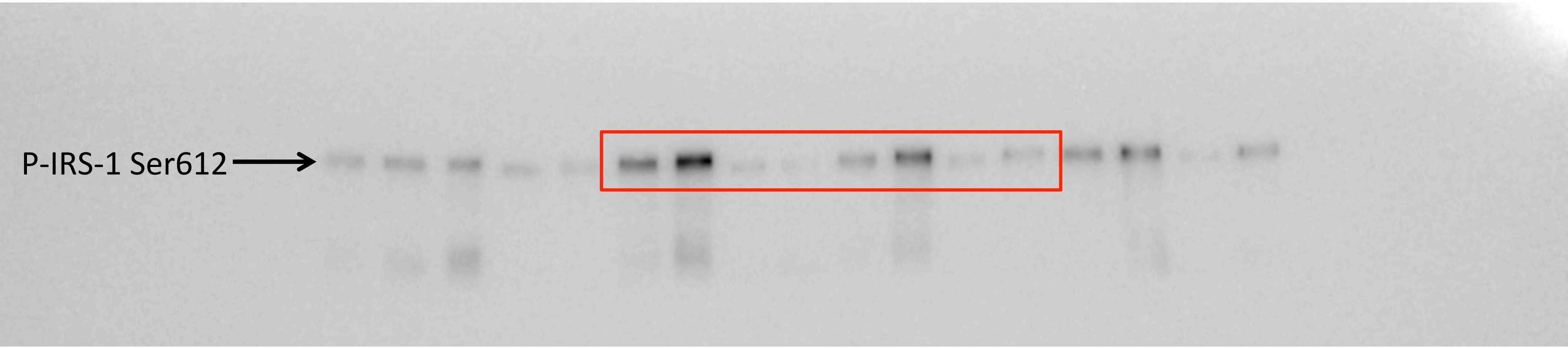

T-IRS-1

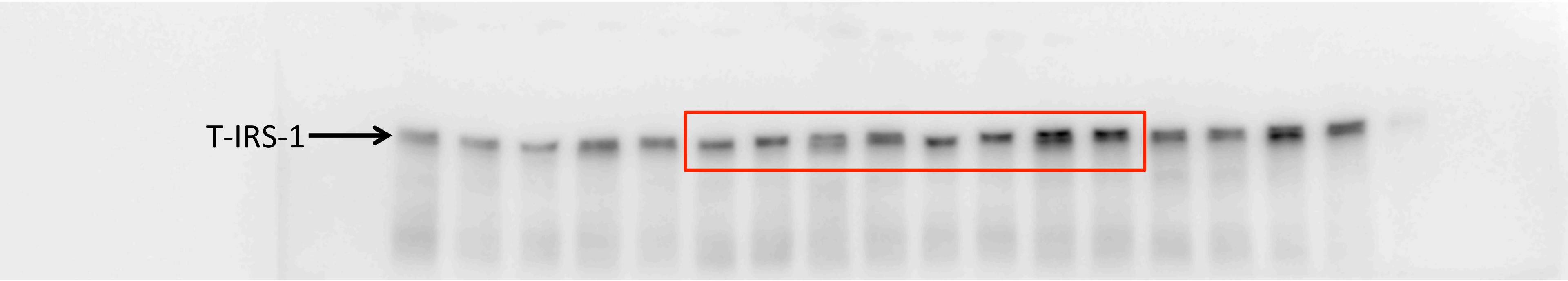

Enhanced skeletal muscle insulin sensitivity after acute resistance-type exercise is upregulated by rapamycin-sensitive mTOR complex 1 inhibition

Kohei Kido, Kohei Sase, Takumi Yokokawa, Satoshi Fujita

Raw blots related to Figure 7

P-Akt Thr308

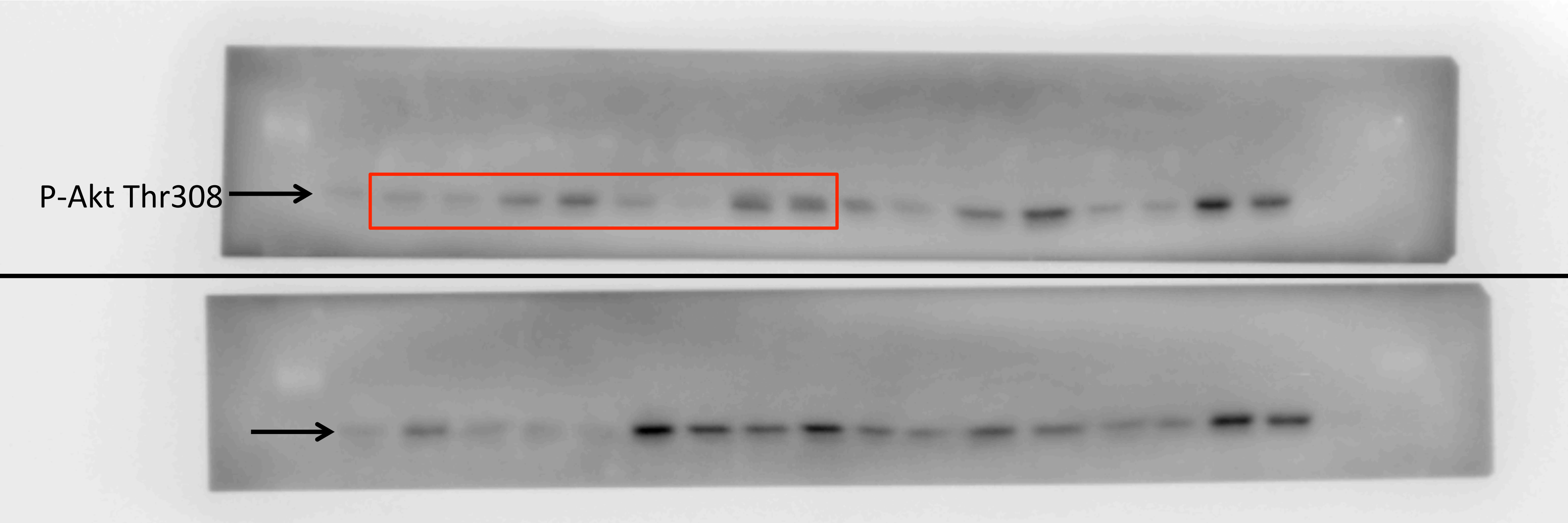

P-Akt Ser473

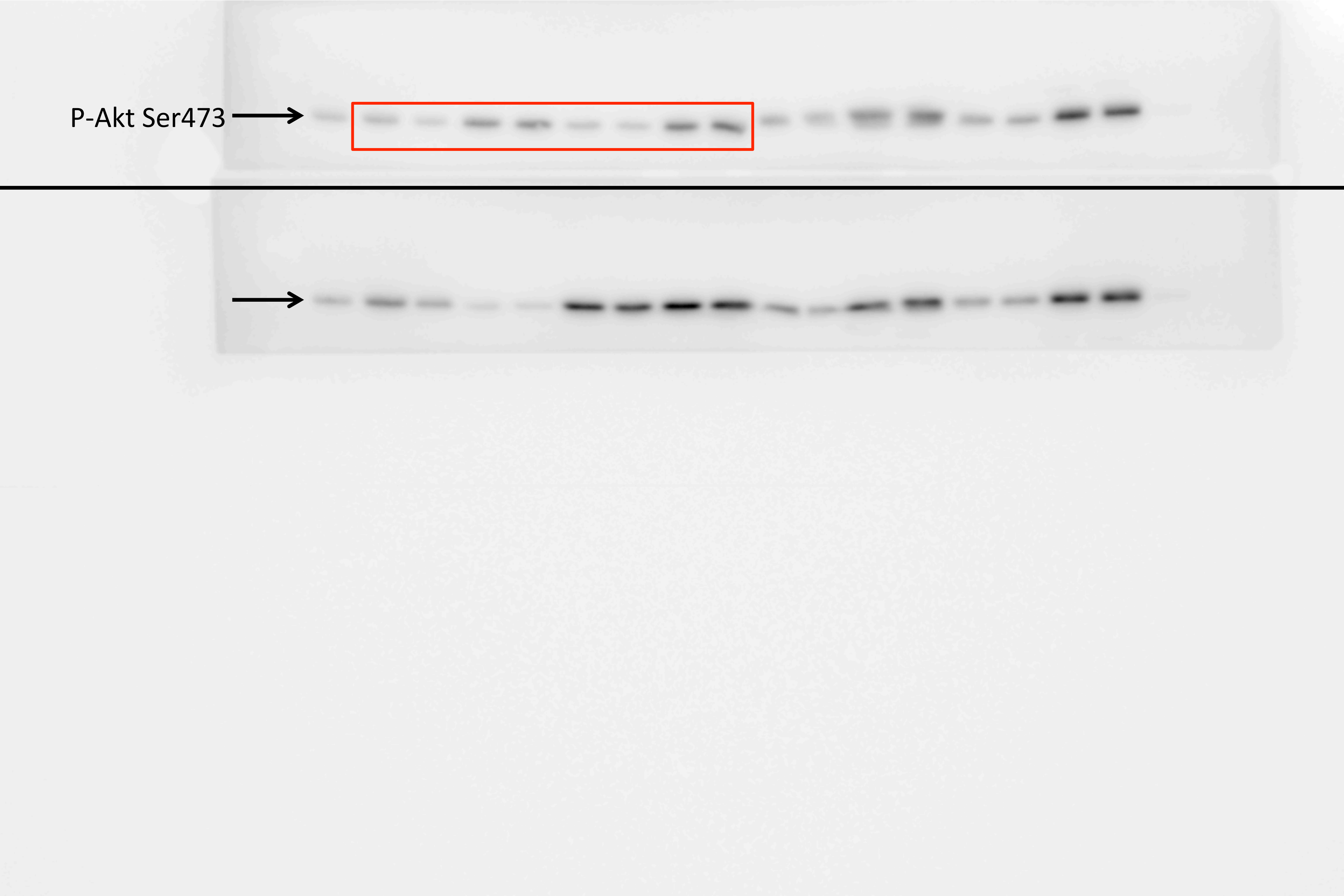

T-Akt

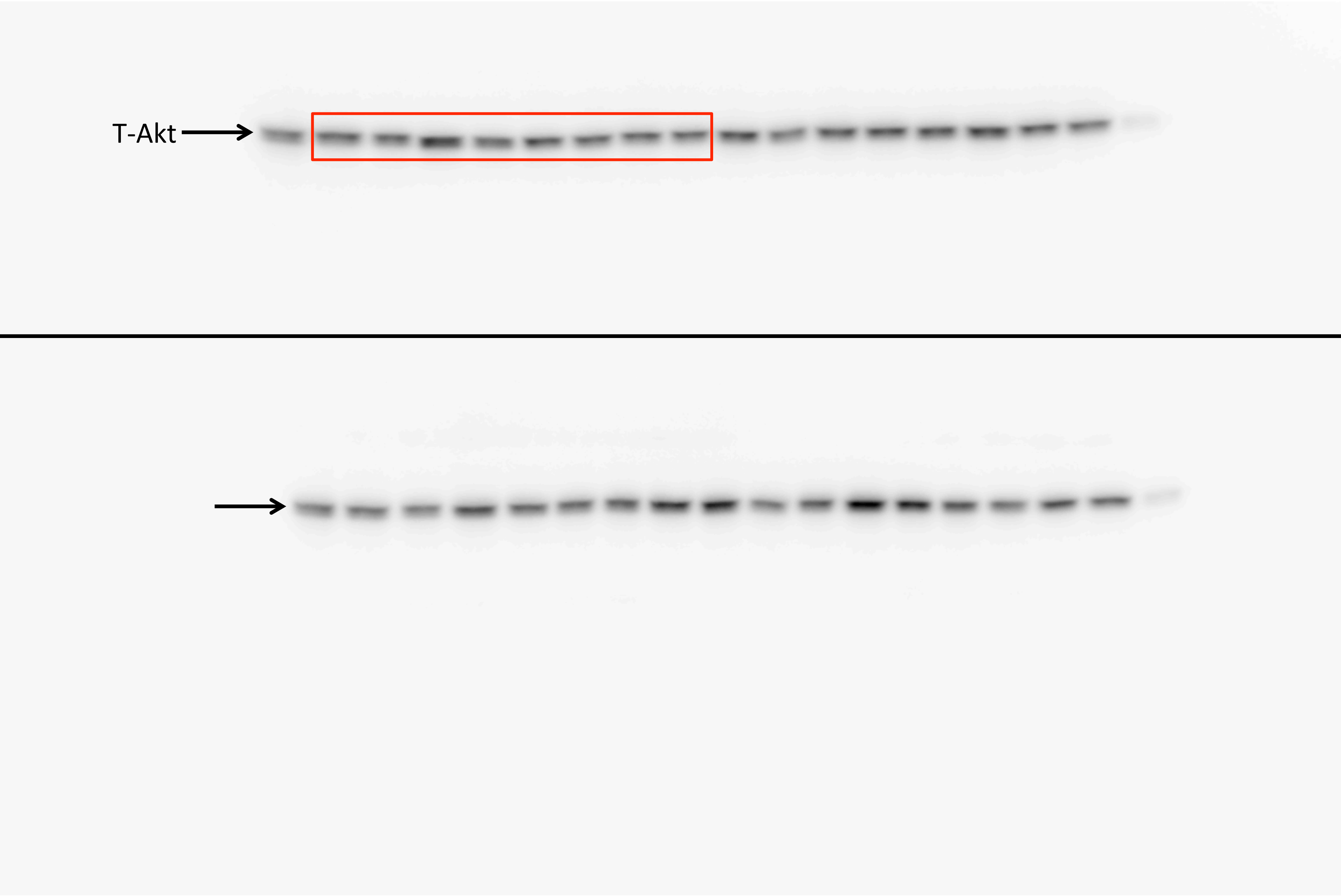

P-TBC1D1 Ser231

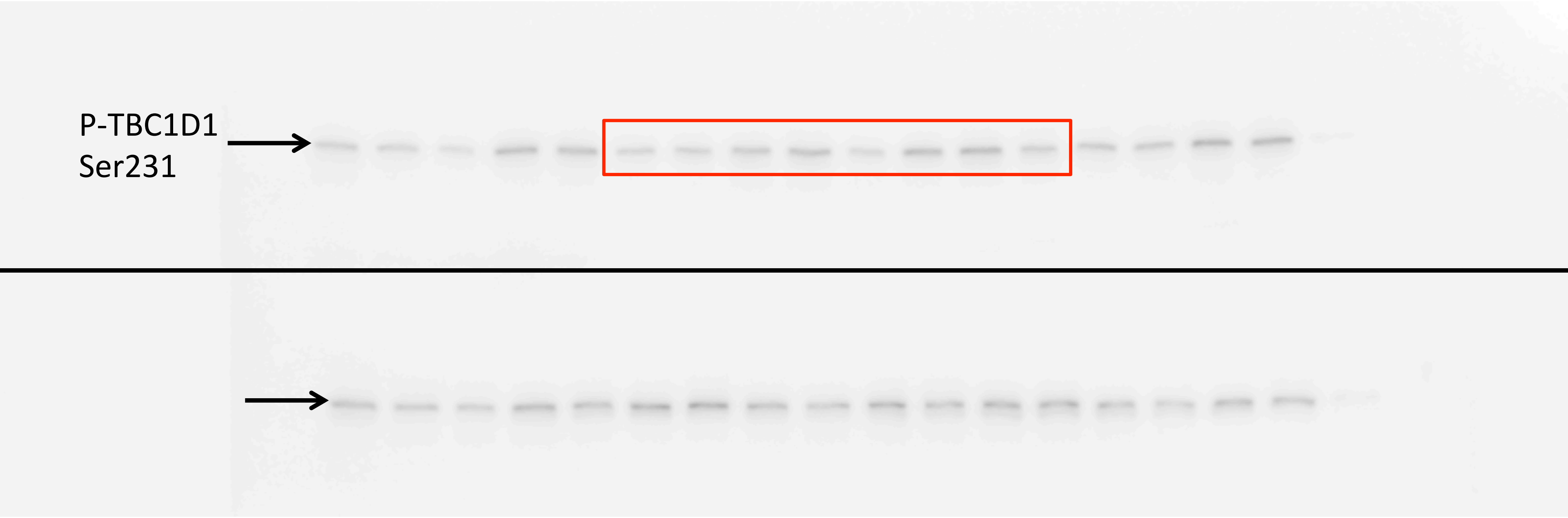

T-TBC1D1

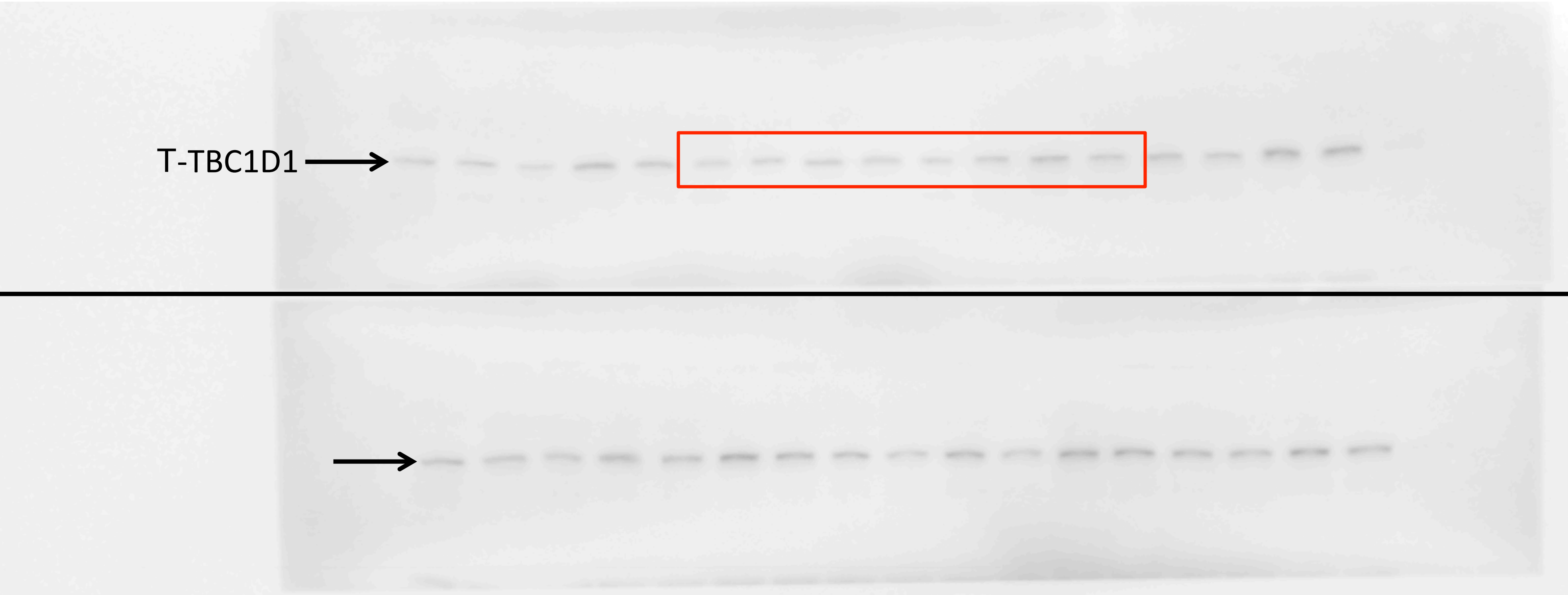

P-TBC1D4 Ser597

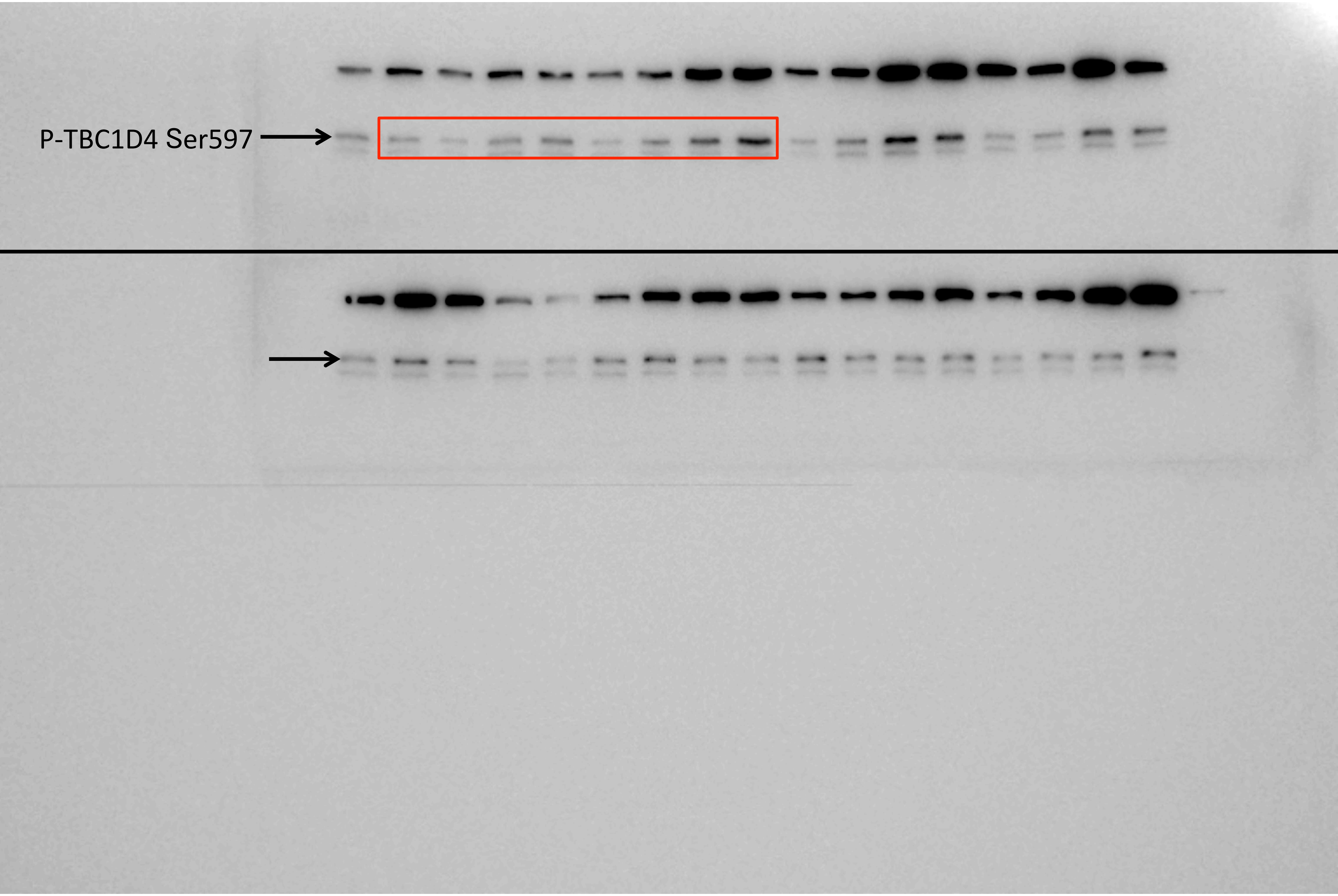

P-TBC1D4 Thr651

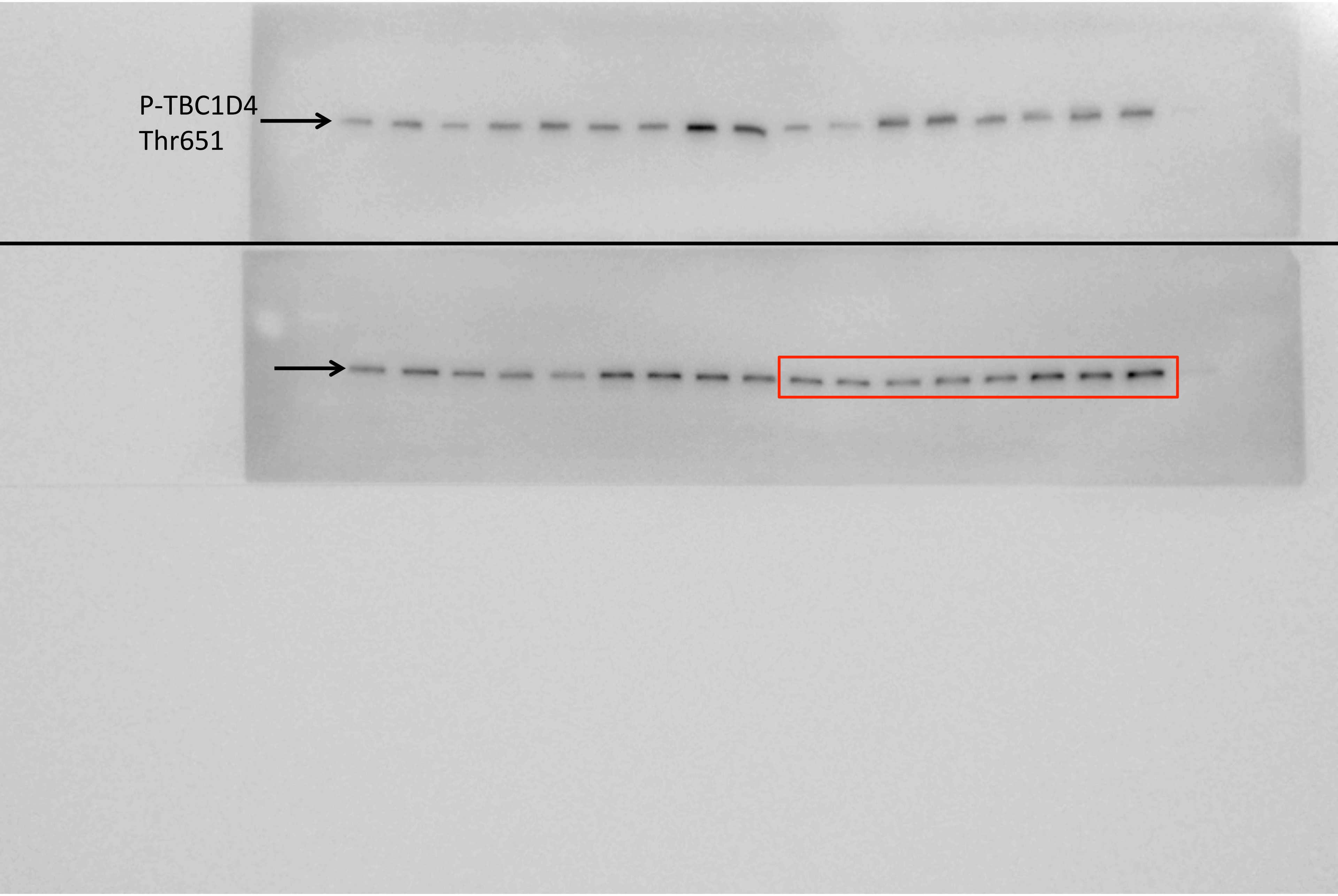

T-TBC1D4

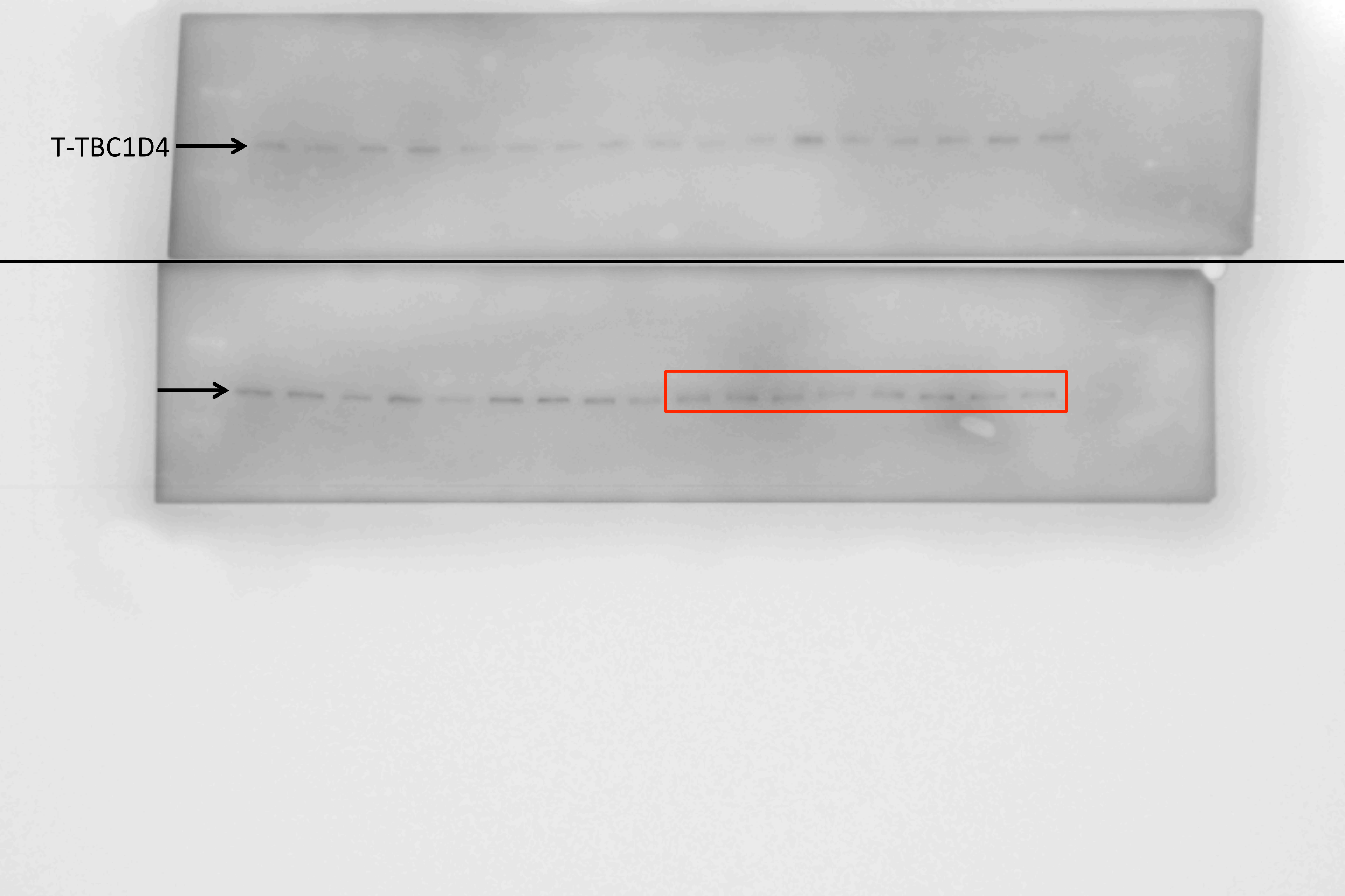

Supplement: Supplementary file 1 — Supplementary information. [file 41598_2020_65397_MOESM1_ESM.pdf]
